# Carbohydrate metabolism in *Oenococcus oeni*: a genomic insight

**DOI:** 10.1186/s12864-016-3338-2

**Published:** 2016-12-01

**Authors:** Alice Cibrario, Claire Peanne, Marine Lailheugue, Hugo Campbell-Sills, Marguerite Dols-Lafargue

**Affiliations:** 1 University of Bordeaux, ISVV, EA 4577, Oenologie, F-33140 Villenave d’Ornon, France; 2 Bordeaux INP, ISVV, EA 4577, Oenologie, F-33140 Villenave d’Ornon, France

**Keywords:** *Oenococcus*, Comparative genomics, Carbohydrate, Metabolism, Wine, Adaptation

## Abstract

**Background:**

*Oenococcus oeni* is the bacterial species that drives malolactic fermentation in most wines. Several studies have described a high intraspecific diversity regarding carbohydrate degradation abilities but the link between the phenotypes and the genes and metabolic pathways has been poorly described.

**Results:**

A collection of 41 strains whose genomic sequences were available and representative of the species genomic diversity was analyzed for growth on 18 carbohydrates relevant in wine. The most frequently used substrates (more than 75% of the strains) were glucose, trehalose, ribose, cellobiose, mannose and melibiose. Fructose and L-arabinose were used by about half the strains studied, sucrose, maltose, xylose, galactose and raffinose were used by less than 25% of the strains and lactose, L-sorbose, L-rhamnose, sorbitol and mannitol were not used by any of the studied strains. To identify genes and pathways associated with carbohydrate catabolic abilities, gene-trait matching and a careful analysis of gene mutations and putative complementation phenomena were performed.

**Conclusions:**

For most consumed sugars, we were able to propose putatively associated metabolic pathways. Most associated genes belong to the core genome. *O. oeni* appears as a highly specialized species, ideally suited to fermented fruit juice and more specifically to wine for a subgroup of strains.

**Electronic supplementary material:**

The online version of this article (doi:10.1186/s12864-016-3338-2) contains supplementary material, which is available to authorized users.

## Background

*Oenococcus oeni* is considered the bacterial species most fitted to the particular conditions of winemaking [1, 2]. Indeed, wine undergoing the malolactic fermentation (MLF) is its main known ecological niche [3, 4], followed by cider and other fermented fruit [3]. In wine, it ferments the residual carbohydrates left by the yeasts at the end of the alcoholic fermentation and transforms malic acid into lactic acid [3]. The degradation of malic acid is described to begin when the lactic bacteria population reaches 10^6^ cfu/ml of wine [4]. The success of spontaneous MLF is therefore linked to the ability of indigenous *O. oeni* to previously grow by using carbohydrates. *O. oeni* is heterofermentative [2–5]. Glucose and fructose, its most studied growth substrates, are oxidized via the phosphoketolase pathway that leads to the excretion of lactate and ethanol (or lactate and acetate, depending on the redox potential of the growth medium) [5–8]. Many carbohydrates others than D-glucose and D-fructose are present in the wine, at low concentrations, at the end of alcoholic fermentation: trehalose, mannose, cellobiose and β-glucosides, L-arabinose, D-xylose, D-galactose and, sometimes, L-rhamnose, D-mannitol, D-sorbitol, melibiose, maltose, lactose or raffinose and sucrose [9, 10]. Complex oligosaccharides and polysaccharides are also present [9, 11, 12].

Many studies have described that *O. oeni* strains displayed quite variable phenotypes regarding the carbohydrate they can use as single growth substrate [2, 5, 13–17]. The degradation of glucose and ribose is generally considered as a general trait in the species. On the other hand, the strains are described to differ regarding the metabolism of fructose, galactose, mannose, arabinose, xylose, trehalose, sucrose, lactose, maltose and melibiose [5, 15–17]. The growth on xylose and/or arabinose was proposed as a test for strain classification by Peynaud and Domercq [13].

Whole genome sequencing projects enabled a more rigorous analysis of *O. oeni* metabolic performance and genetic diversity. The first genome was produced in 2005 and revealed that 10% of the annotated genes were dedicated to carbohydrate metabolism [18] and that 4 to 5% were dedicated to carbohydrate transport [19]. However, the authors underlined the difficulty to make links between genes and phenotypes. More recently, the analysis of the genome sequence of 14 *O. oeni* strains revealed that most of the genes annotated as involved in carbohydrate metabolism formed part of the core genome but that strain specific traits also exist [20]. However, this study did not combine the genomic analysis with phenotyping and the consequences of the genomic variations described remained hypothetical. Meanwhile, Kim et al. [21] identified the genes encoding transport proteins whose expression was induced in the presence of glucose or fructose. Jamal et al. [22] identified the *pts* genes induced in the presence of glucose and fructose but also cellobiose, trehalose and mannose. This last study also pointed out the high degree of *pts* gene conservation in the species, by comparing the 14 genome sequences available at the time.

In a recent paper [23], phylogenomic and population structure analyses over a population of 50 strains revealed a high level of gene synteny conservation. The *O. oeni* species appeared to be divided into several genetic groups of strains. Two major groups of 12 and 37 strains, respectively named A and B, emerged. A putative third group, named C and displaying a single strain was also identified. Group A strains were shown to be predominant in wines. Smaller genetic subgroups, specifically adapted to different products such as Champagne or cider, were proposed and could have been naturally selected through domestication by human activity [23]. Very recently, Sternes and Borneman [24] proposed a pan genome assembly by studying 191 genome sequences and described genes putatively associated with several phenotypic traits, including sugar metabolism but, in the absence of phenotypic or biochemical characterization, the role of these genes remained hypothetical.

To go further, we assessed the diversity of a representative subset of 41 *O. oeni* strains, regarding the ability to grow by using specific carbohydrates, and we then identified the genomic elements that may direct the metabolic activities involved in the phenotypic differences in the 41 studied strains.

## Methods

### Strains

The name and origin of the 41 *O. oeni* strains studied and the accession numbers of the corresponding genome sequences are indicated in Table 1.

**Table 1:** Strains characteristics and phenotyping results

Branch^(1)^	Strain^(2)^	Origin	Glucose	Trehalose	Cellobiose	Ribose	Mannose	Meli biose	L-Arabinose	Fructose	Sucrose	Maltose	Galactose	Xylose	Raffinose	Lactose	L-Sorbose	L-rhamnose	Sorbitol	Mannitol	Total^(3)^	Genome sequence accession number
A	S28^a^	France red wine	1	1	1	1	1		1												6	AZJY00000000
A	S23	England white wine	1	1	1	1	1		1												6	AZLL00000000
A	S11	France white wine	1	1	1	1	1	1	1	1											8	AZJX00000000
A	AWRI_B429^a^	Italy	1	1	1	1	1														5	ACSE00000000
A	Cine^a^	NA	1	1	1	1	1	1													6	AZJV00000000
A	IOEB_0608	France red wine	1	1	1	1	1		1												6	AZKJ00000000
A	IOEB_9517	France	1	1	1	1	1														5	AZKG00000000
A	IOEB_L65.2	Lebanon red wine	1	1	1	1	1														5	AZLR00000000
A	AWRI_B419^a^	France	1	1	1	1	1	1	1												7	ALAF00000000
A	VF^a^	France	1	1	1	1	1	1	1												7	AZLM00000000
A	IOEB_S436a	NA	1	1	1	1	1	1	1												7	AZLS00000000
A	S15	France red wine	1	1	1	1	1	1	1				1								8	AZLJ00000000
A	S161	red wine	1	1	1	1	1	1	1				1								8	AZLN00000000
A	IOEB_S277^a^	France	1	1	1	1	1	1	1												7	AZKD00000000
A	IOEB_L18.3	Lebanon red wine	1	1	1	1	1	1													6	AZLO00000000
A	IOEB_B10	NA	1	1	1		1	1		1											6	AZJW00000000
A	S19	France red wine	1	1	1		1	1		1											6	AZLK00000000
A	IOEB_1491	France red wine	1	1	1		1	1		1											6	AZLG00000000
A	AWRI_B129	France	1	1	1	1	1	1		1											7	AJTP00000000
A	IOEB_L40.4	Lebanon red wine	1	1	1	1	1	1	1	1											8	AZLQ00000000
A	IOEB_S450^a^	France	1	1	1	1	1	1													6	AZLT00000000
A	PSU-1^a^	USA red wine	1	1	1	1	1	1	1	1											8	CP000411 (18)
A	S14	France red wine	1	1	1	1	1	1													6	AZLI00000000
A	B16^a^	France Champagne	1	1	1	1															4	AZKC00000000
A	IOEB_0205	France Champagne	1	1	1	1															4	AZHH00000000
A	AWRI_B548^a^	France Champagne	1	1	1	1															4	ALAH00000000
A	IOEB_0607	France red wine	1		1	1		1													4	AZKK00000000
A	S25	France red wine	1	1	1			1													4	AZJZ00000000
A	IOEB_L26.1	Lebanon red wine	1	1	1	1	1	1						1							7	AZLP00000000
B	S13	France red wine	1	1		1	1	1	1	1											7	AZKB00000000
B	S12	France white wine	1	1		1	1	1	1	1	1	1									9	AZLH00000000
B	IOEB_0501	France red wine	1	1	1	1	1	1	1	1	1	1	1		1						12	AZIP00000000
B	IOEB_0502	France red wine	1	1	1	1	1	1	1	1	1	1		1	1						12	AZKL00000000
B	ATCC_BAA-1163	France red wine	1	1	1	1	1	1		1	1										8	AAUV00000000
B	IOEB_9803	France	1	1	1	1	1	1	1	1	1	1									10	AZKF00000000
B	IOEB_9805	France	1	1	1	1	1	1	1	1	1	1									10	AZKE00000000
B	IOEB_8417	France	1	1	1	1	1	1	1	1	1										9	AZKH00000000
B	IOEB_9304	France cider	1	1	1	1	1	1	1	1		1									9	AZKI00000000
B	IOEB_C28	France cider	1	1	1	1	1	1	1	1			1								9	AZLE00000000
B	IOEB_C23	France cider	1	1	1	1	1	1	1	1	1	1									10	AZJU00000000
C	IOEB_C52	France cider	1	1	1	1	1	1	1	1	1	1		1							11	AZLF00000000
		total/41	41	40	39	37	36	32	23	19	9	8	4	3	2	0	0	0	0	0		
		Total (%)	100%	98%	95%	90%	88%	78%	56%	46%	22%	20%	10%	7%	5%	0%	0%	0%	0%	0%		

^(1)^ The strains are listed according to their phylogenomic proximity and the branch in the dendrogram to which they belong is indicated (see Additional file 2: Figure S2)

^(2)^ The strains proposed and or commercialized as malolactic starters are indicated by^a^

^(3)^ The sugars are classified by order of preference. When a sugar is metabolized by a strain, the number 1 appears in the cell of the table. The number of sugars metabolized by each strain is indicated

### Phenotype characterization

The bacteria were propagated in a semi defined medium (SMD) containing : casamino-acids 10 g.l^−1^, sodium acetate 3.4 g.l^−1^, KH_2_PO_4_ 1 g.l^−1^, MgSO_4_, 7H_2_O 0.1 g.l^−1^, MnSO_4_, 4H_2_O 0.1 g.l^−1^, ammonium citrate 2.0 g.l^−1^, bactotryptone 5 g.l^−1^, yeast nitrogen base 6.7 g.l^−1^, adenine, uracyl, thymine, guanine 5 mg.l^−1^ each, and 10 g.l^−1^ of a single carbohydrate. Eighteen carbohydrates were used for phenotyping: D-glucose, D-fructose, D-ribose, D-xylose, L-arabinose, D-mannose, D-galactose, L-sorbose, L-rhamnose, D-mannitol, D-sorbitol, sucrose, trehalose, cellobiose, melibiose, lactose, maltose and raffinose. The carbohydrate solutions were prepared as 50 g.l^−1^ solutions and were sterilized for 20 min at 121 °C, while the remaining ingredients (base) were prepared as a 2X solution which was sterilized by filtration (0.2 μm cut off). Before sterilisation, the pH was adjusted to 5.5.

The *O. oeni* strains were kept frozen at −80 °C. They were first grown in SMD-glucose medium at 25 °C without agitation. These SMD-glucose grown cells were centrifuged (9 000 × *g*, 4 °C, 5 min), washed with NaCl 9 g.l^−1^ and re-suspended in SMD base 2X in order to obtain an OD_600_ (absorbance at 600 nm in a 1-cm-path cuvette) equal to 1. The cellular suspension was then used to inoculate (10%) 2 millilitres of SMD medium containing the tested carbohydrate. Tubes containing either uninoculated medium with carbohydrate or inoculated medium without carbohydrate were prepared as controls. All the tests were made in triplicate using independent pre-cultures.

After a 2-week-incubation, the OD_600_ of the cell suspension was measured. Two hundred microlitres of the cell suspension were mixed with 40 μl of Bromocresol green (0.25 g.l^−1^ in aqueous solution) in a microplate and the color change (blue to green or even yellow) was estimated with the naked eye. The culture supernatant was collected after centrifugation (10 000 × *g*, 4 °C, 5 min) and kept at −20 °C for HPLC analysis.

Carbohydrates, lactic acid, acetic acid, glycerol and ethanol concentrations in the culture supernatant were measured by anion exchange chromatography (Aminex HPX87H column, Bio-Rad) using a Waters (Milford, USA) system consisting of a pump (Waters 600), an injector (Waters 717) and a refractometer (Waters 2414). The eluent (H_2_SO_4_ 5 mM) had a constant flow rate equal to 0.5 mL.min^−1^, at room temperature. For fructose and mannitol assays, a Aminex HPX87K column was eluted with K_2_HPO_4_ 10 mM, 0.5 mL.min^−1^ at 65 °C.

A significant colour change (from blue to green or yellow), associated with a production of lactate higher than 10 mM, was considered as a positive phenotype, while assays leading to lactate production lower than 5 mM were always associated with no colour change and were considered negative. No intermediate situations were observed. Most of the time, the OD_600_ change was correlated with the color and HPLC results, but sometimes, slight OD_600_changes (+0.2 OD_600_ unit) were observed in the absence of carbohydrate degradation. These tests were considered negative, as proposed by Hocine et al. [15].

For growth-rate determination, 50 ml vessels containing SMD-glucose were inoculated, incubated at 25 °C, and OD_600_ measurements were performed daily during 2 weeks.

### Genome screening, gene identification and nomenclature

The genome sequencing and annotation methods and the genome features have been described by Dimopoulou et al. [25] and Campbell Sills et al. [23]. Publicly available *O. oeni* and *O. kitaharae* genomes associated with previous work were also used [18, 20, 26, 27]. Phylogenomic comparisons were carried out using ANIm algorithm [28] which generated a distance matrix [23] that was then used to construct a tree, restricted to the 41 strains, by the neighbor joining method with MEGA v7 [29].

All the coding sequences in all the genome sequences were annotated with RAST [30], which also enabled to compare the genomes two by two. Genome comparisons were also done using orthoMCL v2.0.9, which enabled to define the core genome (restricted to the 41 strains studied). To further assign predicted protein functions, we used either the Pfam database (http://pfam/xfam.org), the CAZy database (www.cazy.org/), or, for transport proteins, the TCDB database and the ABCdb database (http://www.tcdb.org/; https://www-abcdb.biotoul.fr/). Analysis also included BLASTp comparisons with non-redundant protein databases (http://www.ncbi.nlm.nih.gov/Blast.cgi) [31].

### Signal peptide

The presence of signal peptides in the 5’ end of deduced protein sequences was analyzed through signalP 4.1 software (http://www.cbs.dtu.dk/services/SignalP/).

### Correlations

In order to find out correlations between phenotypes (either positive or negative) and genome sequences properties, two distinct methods were used. For each sugar considered, a subset of positive and negative strains was constituted and a list of genes specifically present/absent in positive strains genome sequences was searched, with no a priori regarding the gene annotation and associated protein function. Correlations were statistically evaluated using ape 3.4 R-package.

On the other hand, a list of genes putatively associated to the degradation of each sugar considered according to gene annotation was established. The list could be particularly long regarding the transporters putatively associated with the sugar transport as the specificity prediction is often difficult for such proteins. Sequence comparisons were made with BLAST and RAST [30, 31] and mutations specific for positive or negative strains were examined.

From the genomes sequences and the correlations results, we created a database of protein sequences, putatively associated with each carbohydrate metabolism (Additional file 1: Table S1).

## Results and discussion

A panel of 41 *O. oeni* strains was studied. The strains were chosen for the diversity of their origin (country, winemaking region and type of product) and for their respective position on the species phylogenomic dendrogram, in order to make a description as representative as possible of the species diversity. The dendrogram is described on Additional file 2: Figure S2: twenty nine strains belong to branch A, which is generally described as gathering the domesticated strains [23, 32, 33], eleven strains belong to branch B, which is generally described as gathering strains more “wild” than those in branch A and a single strain belongs to branch C. From the evolution point of view, this strain was suggested to form part of a group that preceded groups A and B in which a lot of genetic functions were lost [23]. Three cider strains and 3 Champagne strains are grouped onto two specific branches on the dendrogram.

### Phenotyping results

The phenotypes obtained are reported in Table 1. None of the 41 strains was able to use all of the eighteen carbohydrates studied. On average, the strains metabolized about 7 sugars, but a high diversity was observed: six strains metabolized 10 or more sugars, 31 strains metabolized between 5 and 9 sugars and 5 metabolized only 4 of the 18 sugars studied.

The most consumed carbohydrates were glucose (consumed by all strains), followed by trehalose, consumed by 98% of strains, cellobiose, ribose and mannose (more than 88% of the strains), melibiose (over 75% of strains) and arabinose and fructose (about half of the strains). Then, there were substrates consumed by a minority of strains: maltose or sucrose (consumed by 20% of the strains), galactose, raffinose and xylose (consumed by 5 to 10% of the strains). None of the studied strains was able to grow on lactose, mannitol, sorbitol, L-rhamnose or L-sorbose.

The strains in branch A were less versatile as, on average, they were able to grow on 6 of the carbohydrates studied when those in branches B were able to grow on about 10 sugars. The single strain in branch C metabolized 11 of the sugars studied. All the strains belonging to the branches B and C metabolized glucose, trehalose, ribose, mannose, melibiose and fructose and a large majority of strains in these branches also metabolized L-arabinose, cellobiose, maltose and sucrose. Conversely, cellobiose and glucose were consumed by all strains in branch A. The strains in branch A also frequently consumed trehalose, ribose, mannose, and melibiose. Most of the time, strains in the same “genomic subgroup” according to Additional file 2: Figure S2 often displayed the same phenotypes : this was the case with strains IOEB_1491 and S19 or strains IOEB_9517 and IOEB_L65_2 or even, the three Champagne strains in branch A. However, there were also cases where proximal strains showed distinct phenotypes: this was the case of the three following trios of strains :S15, S161 and IOEB_S277, S28, CiNe and AWRIB-429, or the 3 cider strains in branch B (Table 1).

Among the strains studied, some have been or are marketed as malolactic starters (CiNe, AWRIB_429, S28, IOEB_S450, PSU-1 AWRI_B548, AWRI_B419, VF, B16 and IOEB_S277). These strains metabolized from 4 to 8 of the substrates studied.

### Metabolic pathway reconstruction

**Fig. 1:**
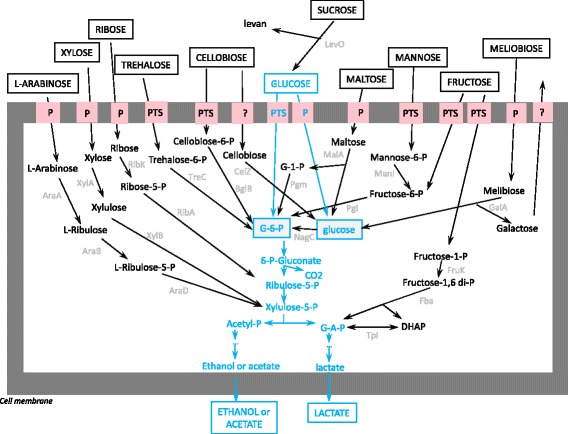
Schematic representation of the metabolic pathways identified in *O. Oeni*. The catabolic pathways for glucose degradation appear in *blue*, because they have been at least partly characterized at the biochemical level in *O. oeni* [21]. The pathways identified through the genotype/phenotype correlations in this study appear in *black*, with the short enzymes names in *gray*. The transporters are indicated: PTS indicates a PTS permease, while P indicates a non PTS transporter that can be a MFS permease or an ABC transporter, and ? an unidentified uptake or efflux system. The co substrates such as ATP, NADP(H), PEP are not indicated. Other sugar catabolic pathways are encoded in the genomes. The correlations being less clear, they are not shown in this figure and will be described more precisely in the text. *When the series (D, L) is not specified, the monosaccharides belong to D series. When several proteins can ensure the same function (ex. 3 NagC isoforms were found), a single name is indicated*

Figure 1 gathers the most probable degradation pathways for D-glucose, D-fructose, D-mannose, D-xylose, D-ribose, L-arabinose, cellobiose, trehalose, sucrose, maltose and melibiose in *O. oeni*. These routes were validated through genome analysis (presence of the gene encoding the associated enzymes and transporters) and through genotype/phenotype correlations. The pathways for glucose catabolism are the only ones that had been confirmed through biochemical studies [21]. The others remain hypothetical. Some of them (fructose, mannose, arabinose and xylose) have already been suggested by genome survey [18, 20, 24]. We analyzed their relevance sugar by sugar, using phenotypic and genomic results and any other relevant result available in the literature. Alternative pathways or pathways for other carbohydrate degradation are proposed in this paper but, as the genotype/phenotype correlations are less clear, they are not presented in Fig. 1. All the pathways described converge with the glucose catabolic pathway at a specific point that can be glucose-6-P, ribulose-6-P, xylulose-5-P or glyceraldehyde-3-P. Only the biochemical steps upstream of these intermediates are discussed in this paper. These steps include 1/sugar transport into the cell, 2/hydrolysis in the case of a glycoside and 3/phosphorylation and isomerization. The 3 steps can occur in this order or in a different one, depending on the carbohydrate considered.

The genes encoding functions associated with carbohydrate metabolic pathways described in this study were all found on the chromosome of the bacteria.

### Glucose, fructose and mannose

**Fig. 2:**
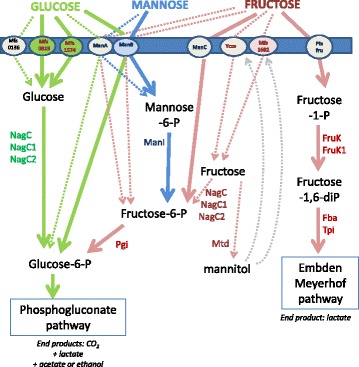
Putative pathways for transport, phosphorylation and isomerisation of glucose (*green*), mannose (*blue*) and fructose (*pink*). The full lines describe the permeases such as Mfs1574 and Mfs0819 whose activity was demonstrated by heterologous gene expression [21] or the pathways for which good correlations were obtained in this study. The other pathways are described by dotted lines because the links between genotypes and phenotypes are less clear. NagC NagC1, NagC2: hexokinases; ManI: phosphomannose isomerase; Pgi : phosphoglucose isomerase; Mtd: mannitol deshydrogenase; FruK: 1-phosphofructokinase, Fba: fructose-bisphosphate aldolase; Tpi: triose phosphate isomerase; Pts^fru^: four distinct operons (*fruA*, *fruB*, *fruC* and *fruD*) encode four distinct PTS permease sets. ManA, ManB, ManC: PTSmannose permease; Ycze and MfsXXXX, MFS permeases. The co substrates such as ATP or PEP are not indicated

**Fig. 3:**
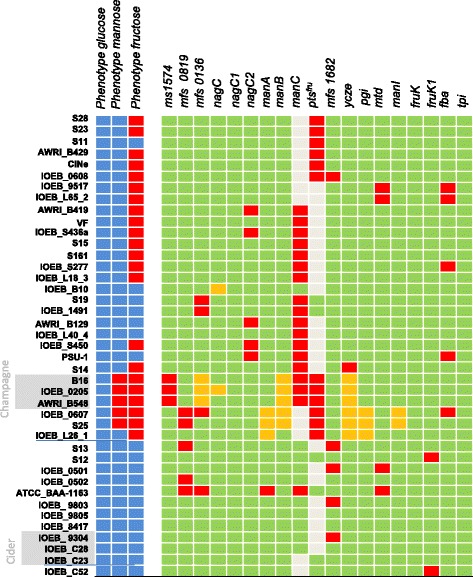
Genotype/phenotype correlations regarding glucose, mannose and fructose degradation. Strains appear in the same order as on the phylogenomic dendrogram (Additional file 2: Figure S2). In the lane for phenotypes, the *blue color* indicates that the strain is able to grow on glucose, fructose or mannose as the sole carbon source, and the *red color* indicates that the strain is unable to grow in such conditions. In the lanes for genotypes, a *beige box* indicates that the gene or the operon is absent. A *red box* indicates that the gene or one of the genes in the cluster (such as the *pts* operons *manA*, *manB*, *manC* and *pts* ^*fru*^) is truncated or appears as a pseudogene. The *green color* indicates no gene truncation but mutations still can lead to inactive proteins. An *orange box* indicates that the corresponding genome sequence displays a specific gene mutation leading to a singular protein. The gene nomenclature is indicated in Fig. 2

We simultaneously examined the pathways for glucose, fructose and mannose transport and phosphorylation (Fig. 2) and genotype to phenotype correlations were carried out (Fig. 3). However, the fact that all the studied strains were glucose consumers makes the analysis less robust with this specific sugar.

Most of the genes putatively involved in the glucose catabolic pathways (according to associated protein sequence analysis, Additional file 1: Table S1) belong to the core genome restricted to the 41 strains studied: three distinct MFS permease genes *mfs0819*, *mfs1574* and *mfs0136,* three hexokinases genes (*nagC*, *nagC1* and *nagC2*) and two *pts* operons *manA* and *manB*. The protein NagC is generally annotated as a glucokinase, while NagC1 and NagC2 are annotated as fructokinases, although they display the same PFAM classification and none has been characterized at the biochemical level for substrate specificity. No *pts* ^*glc*^ operon is found in any of the 41 genomes studied. A third *pts* mannose operon, *manC,* is present in 30 of the genome studied but in a truncated form in twenty strains (Fig. 3). The gene *mfs1574* is a pseudogene in 3 of the 41 strains studied, while *mfs0819* is a pseudogene in 5 other strains. In all the strains examined, among these two genes, there is always at least one encoding an apparently active permease. The gene *mfs0136* is somewhat less conserved but encodes a putative additional permease in 38 strains. NagC is 100% conserved in 39 of 41 strains and displays a mutation outside the catalytic site in the two remaining strains (IOEB_B10 and IOEB_0205). NagC1 is 100% conserved while *nagC2* is a pseudogene in 5 of the studied strains. And, most of the time, at least one of the PTS operons *manA*, *manB* or *manC* encodes a potentially active PTS permease. As a result, there is always at least one of the routes described in Fig. 2 potentially active, which is in accordance with the fact that all studied strains metabolized glucose (Fig. 3). The genes *mfs0819* and *mfs1574* were shown to be highly overexpressed during cultures of *O. oeni* B1 (no genome available) on glucose, and the glucose uptake activity of the encoded proteins was demonstrated by heterologous expression in *Bacillus subtilis* [21]. The gene *mfs0136* was slightly overexpressed in the presence of glucose, but the activity of the encoded protein has not been demonstrated [22]. The *manB* operon was shown to be overexpressed during cultures on glucose and fructose [21, 22]. In Champagne strains, which exhibit a particularly slow growth compared to the others (μ ≈ 0.35 day^−1^ against 0.6–0.7 day^−1^ for most strains), several genes associated with glucose catabolism seem singular: the gene *mfs1574* is truncated while Mfs0136 and ManB display specific protein sequences. On the contrary, in strain ATCC BAA-1163, which present a very high growth rate on glucose medium (μ ≈ 0.8 day^−1^), the catabolic routes through Mfs1574 + hexokinase or through ManB were the only potentially active ones. These results and the literature thus suggest that ManB and Mfs1574 + hexokinase are the most active routes for glucose uptake and phosphorylation. However our results do not exclude the other routes described in Fig. 2.

According to genome and protein sequence analysis, two permeases are potentially associated with fructose uptake (Fig. 2, Additional file 1: Table S1): Mfs1682 and Ycze. The gene *ycze* is located in a gene cluster highly conserved among lactic acid bacteria and especially in the genus *Oenococcus* (Additional file 3: Figure S3). This cluster –that will be named the fructose gene cluster in this study- also comprises the fructokinase gene *nagC1*, and a mannitol dehydrogenase gene *mtd*. The role of the *pts* mannose operons, *manA, manB* and *manC* already described for glucose uptake was examined together with that of the *pts* ^*fru*^ operons found in a limited number of strains. Four distinct *pts* ^*fru*^ clusters are found, with distinct gene content and chromosome insertion site: *fruA*, *fruB*, *fruC* or *fruD* (Additional file 1: Table S1).

Less than half of the strains studied were able to grow on fructose: all the strains in branch B and C and specific strains in branch A. Genotype to phenotype correlations show no link with the pathway going through permeases and NagC, NagC1 and/or NagC2 kinases, nor through ManA or ManB, although *mfs1682 and manB* are described to be overexpressed during *O. oeni* growth on fructose [21]. On the other hand, all the strains that display a complete and putatively functional ManC or PTS^fru^ permease were positive. This could explain 15 out of the 19 positive phenotypes. PTS^man^ produces Fructose-6-phosphate, while PTS^fru^ produces Fructose-1-phosphate (Fig. 2). This supposes that part of these strains (IOEB_B10, S13, IOEB_0501, IOEB_0502, IOEB 9803, IOEB_9805, IOEB_8417, IOEB_9304 and IOEB_C28), that display active ManC, assimilate fructose into fructose-6-P which is then converted to glucose-6-P to enter the phosphogluconate pathway, while the others (S19, IOEB_1491, ATCC_BAA1163, IOEB_C23, IOEB_C52) assimilate fructose into Fructose-1-P through PTS^fru^. Fructose-1-P then enters the Embden-Meyerhof pathway. Both pathways (through Fructose-6-P and through Fructose-1-P) may be active in strain S12. This supposes that a homolactic behavior, with lactate as the main end product, may be observed with certain *O. oeni* strains growing on fructose. This was confirmed through HPLC analysis for strain S19. For 1 mmol of fructose consumed, *O. oeni* S19 produced 1.5 mmol of lactate, 0.2 mmol of acetate and less than 0.1 mmol of mannitol, while strain IOEB_0501 produced 0.5 lactate, 0.6 acetate and 0.5 mannitol). Genotype to phenotype correlations do not enable finding a specific route, active in the four remaining positive strains (S11, IOEB_L40.4, AWRI_B129 and PSU-1), and inactive in the negative strains. The role of the permeases Mfs1682 an Ycze and of kinases NagC, NagC1 and NagC2 remains unclear. The mannitol forming activity has been described as a general trait in *O. oeni* [2, 5–7] and was confirmed with strains S19 and IOEB_0501. Mtd appears as an intracellular protein (no signal peptide), and the cells thus need to import fructose and then export mannitol to display this activity; Ycze and Mfs1682 could thus contribute to mannitol formation for either the uptake of fructose or the export of mannitol. However, this necessitates that NagC, NagC1 and NagC2 are not or are very slow fructokinases to be compatible with all the fructose negative phenotypes.

For mannose uptake and phosphorylation, no alternative to PTS^man^ (gene clusters *manA, manB* or *manC*) appears in the genomes studied. Mannose-6-phosphate is then converted to fructose-6-phosphate by phosphomannose-isomerase (ManI, EC 5.3.1.8). Five strains were unable to grow using mannose as sole carbon source: the strains B16, IOEB_0205, AWRIB_548, S25 and IOEB_0607. The gene *manI* was highly conserved in the 41 studied strains, including those with negative mannose phenotype. The *manA* operon encoded a potentially active and not singular permease in 3 of these 5 negative strains (B16, IOEB_0205 and AWRI_B548), and it was truncated in a positive strain (ATCC_BAA-1163), which makes it unlikely that ManA is involved in mannose uptake. By opposition, there was a singular ManB permease (specific mutation in the IIC subunit) in the 5 mannose negative strains, which suggests that mannose transport could be driven by ManB*,* despite the fact that both *manA* and *manB* are overexpressed in the presence of mannose [22].

### Trehalose

**Fig. 4:**
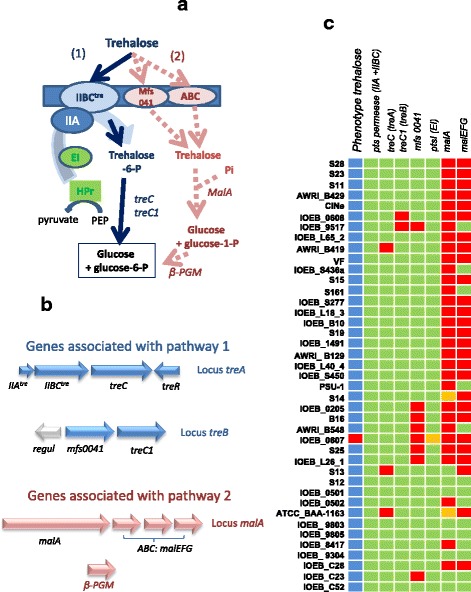
Trehalose degradation. **a** Distinct putative pathways for trehalose transport, phosphorylation and hydrolysis. Pi: inorganic phosphate; PEP: phosphoenolpyruvate; A and IIBC: elements of the PTS permease; Mfs and ABC: sugar transporters. **b** Genes associated with the putative pathways. The genes encoding the general PTS protein (EI and Hpr) of *O. oeni* have been described by Jamal et al. [22]. **c** Genotype/phenotype correlations. Strains appear in the same order as on the phylogenomic dendrogram (Additional file 2: Figure S2). In the lane phenotypes, the blue boxes indicate the strains able to grow on trehalose as the sole carbon source, and the *red ones* indicate the strains unable to grow in such conditions. In the lanes for genotypes, a *red box* indicates that the gene or one of the genes in the operon is truncated or appears as a pseudogene. The *green color* indicates no gene truncation but mutations still can lead to inactive proteins. The *orange box* indicates that the corresponding genome sequence displays a specific mutation leading to a singular protein (i.e., EI is singular in *O. oeni* IOEB 0607). As the genes encoding Hpr and β-PGM are highly conserved, they do not appear in this table

All the strains studied but one (IOEB_0607) were able to grow on trehalose, α-D-glucopyranosyl-1,1-α-D-glucopyranose (Fig. 4). Trehalose may be phosphorylated and transported via a PTS permease to produce trehalose-6- P which is then cleaved by a phosphotrehalase, TreC (Fig. 4a). The activity of this pathway has been demonstrated in PSU-1 strain [22]. The genes associated with this route are grouped in an operon (*treA*). A second phosphotrehalase gene (*treC1*) is found in locus *treB*, next to a MFS permease gene (Fig. 4b and c). A second pathway may be active, in which trehalose is transported by an ABC transporter and then phosphorylated and hydrolyzed by a trehalose phosphorylase, MalA, to generate glucose 1-P and glucose (Fig. 4a). Beta-glucose-1-P would then be converted to glucose-6-P by a β-phosphoglucomutase.

The loci *treA treB* and *malA,* and all the isolated genes putatively involved in trehalose catabolism belong to the core genome (Fig. 4c). The PTS permease in locus *treA* is putatively active in all the strains studied (no mutation or gene truncation). The phosphotrehalase gene in *treA* is truncated in 3 strains without affecting phenotype (ATCC_BAA-1163, S13 and AWRI_B419) which could be explained by complementation with the phosphotrehalase gene in locus *treB*. This first route thus appears as potentially active in all the strains studied, including the negative strain IOEB_0607. However, in this strain, the enzyme EI (involved in the PTS phosphorylation cascade, Fig. 4a) is mutated near the active site, and this could abolish the PTS phosphorylation activity. The pathway through MalA thus appears as complete and potentially active in 8 strains in group B (IOEB_0501, S13, C23, IOEB_9304, S12, C52, IOEB_9803, and IOEB_9805). However, it is difficult to associate clearly this pathway to the metabolism of trehalose because, in all these strains, the first route is also potentially active. Moreover, we will see below that this route is rather associated with maltose degradation. There is no evident link between the MFS permease encoded in *treB* and trehalose degradation.

### Pentose metabolism

We then analyzed the growth of *O. oeni* on 3 pentoses potentially present in wine: D-ribose, L-arabinose and D-xylose.

### D-Ribose

Ribose was the most frequently consumed pentose studied: 90% of the strains used it as a growth substrate. The putative ribose degradation pathway in *O. oeni* is depicted in Additional file 4: Figure S4A. The genes putatively associated with this route are located in 3 distinct gene clusters (Additional file 4: Figure S4B). We found two ribokinase genes (*ribK, ribK1*), two ribose isomerase A genes (*ribA, ribA1*) and 2 permease genes (*ribT*, *mfs148*) potentially associated with ribose uptake. All but *ribT* belong to the core genome. The gene *ribT* was lost in strain IOEB_C23 due to a reorganization of the 3’ end of the ribose1 cluster. The genes *ribK* and *ribA* are pseudogenes in a limited number of strains without altering the ribose phenotype. This suggests that complementation with *ribK1* and *ribA1* is possible in the strains concerned. By opposition, when *ribT* is truncated by mutation, the ribose phenotype of the strain is negative (IOEB_1491, B10 and S25 and S19). The strain C23, which does not display *ribT*, has a positive phenotype that can be explained by the presence, in this strain, of a specific ribose permease gene, *mfs9C23*. The role of the permease encoded in cluster ribose 3, Mfs0148, remains unclear.

### L-Arabinose

**Fig. 5:**
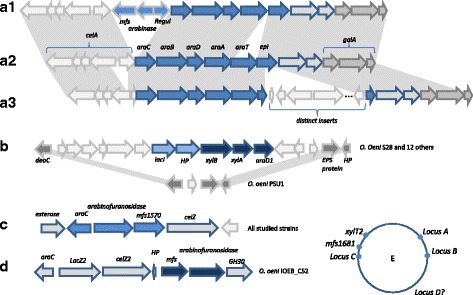
Schematic representation of the various gene clusters putatively involved in arabinose degradation. **a** First arabinose gene cluster, belonging to the core genome, and represented in the 3 distinct versions (**a1**, **a2** and **a3**) found in the genomes studied. **b** Second arabinose gene cluster present in 13 of the studied strains. **c** Third arabinose gene cluster belonging to the core genome. **d** Fourth arabinose gene cluster, present in a single strain. **e** Localization of the various arabinose clusters in *O. oeni* chromosome. *O. oeni* PSU-1 is represented with its own clusters (**a** and **c**) and *mfs* permease genes. The adjacent regions of cluster B enabled to localize it, while the position of cluster D remains unclear, but it should be located on the chromosome as strain IOEB_C52 does not display any plasmid [23]. The genes putatively associated with arabinose catabolism appear as *blue arrows*. AraC: regulator; AraA: L arabinose isomerase; AraB: L-ribulokinase; AraD: L-ribulose-5-P-4-isomerase; AraT: permease. Others putative arabinose proton symporters are found all over the chromosome and are not described here

**Fig. 6:**
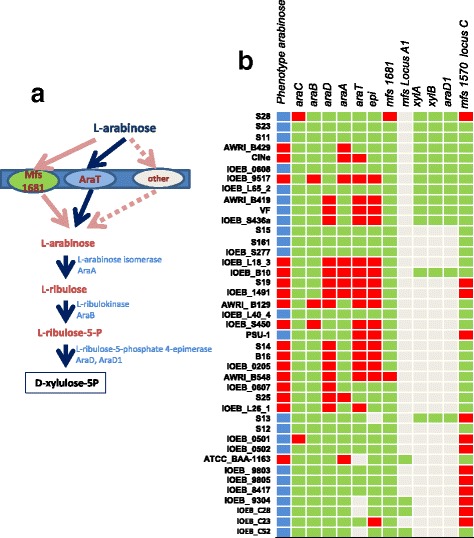
Arabinose degradation. **a** Putative pathway for arabinose transport, phosphorylation and hydrolysis. *AraA: L arabinose isomerase; AraB: L-ribulokinase; AraD: L-ribulose-5-P-4-isomerase; AraT, Mfs, Mfs1681: sugar permeases.* Other putative arabinose transporters are described in Additional file 1: Table S1. **b** Genotype/phenotype correlation. Strains appear in the same order as on the phylogenomic dendrogram (Additional file 2: Figure S2). In the lane describing the phenotypes, a *blue box* indicates a strain able to grow on arabinose as the sole carbon source, and a *red* one indicates a strain unable to grow in such conditions. In the lanes for genotypes, a *beige box* indicates that the gene is absent. The clusters and genes names correspond to those described in Fig. 5. A *red box* indicates that the gene is truncated or appears as a pseudogene. The *green* color indicates no gene truncation but mutations still can lead to inactive proteins

Fifty-six percent of the studied strains were able to grow in the presence of arabinose as the sole carbon source. Several genes putatively associated with arabinose uptake and degradation are found. The first ones are grouped in a specific cluster (Fig. 5a). Most of the genes in this cluster belong to the core genome of *O. oeni* but a significant number of strains display gene deletions and/or insertions and 3 distinct compositions are observed for this cluster (Fig. 5 a1, a2 and a3). The A2 version may be the complete one (*araC, araB, araD, araA, araT* and *epi*), the A1 version has three additional genes encoding a permease, an arabinase and a regulator and the transporter gene *araT* is absent. The A3 version has an insert in the epimerase gene, and this insert differs depending on the strain examined. The arabinose gene cluster A2 appears to encode all the functions necessary for transport, phosphorylation and isomerization of L-arabinose into D-xylulose-5-P, through the pathway depicted in Fig. 6a. Three other gene clusters, located remotely from locus A on the chromosome, also display genes potentially associated with arabinose metabolism (Figs. 5b and 6e). The cluster B is present in 13 strains, the cluster C belongs to the core genome and the cluster D is found only in strain IOEB_C52. The cluster B displays a second isomerase gene *araD1*, next to a gene annotated as encoding an isomerase converting L-xylulose-5-P to D-ribulose-5-P and a gene encoding a carbohydrate kinase (*xylB*) whose substrate specificity cannot be predicted from sequence analysis. Clusters C and D encode permeases and glycoside-hydrolases among which some are annotated as arabinofuranosidases.

The correlations between genotype and phenotype are presented in Fig. 6b. All the strains that display untruncated *araA + araB* + *araD* are able to grow on L-arabinose. Otherwise stated, when *araA*, *araB* or both are truncated (as in strains AWRI_B429, IOEB_S450 or ATCC_BAA-1163), the phenotype is negative, suggesting that no rescue exists in the chromosome. When *araD* is truncated, the phenotype is negative (as in strain IOEB_0607), except when the strain displays *araD1 (*as in strain AWRI_B419).

On the contrary, the 3 genes *araC, araT* or *epi* in cluster A appear non-essential for arabinose degradation. When the predicted protein triad AraA, AraB and AraD appears functional, the absence or the mutation of *epi* (in strains S23, S11, S15, S13 and IOEB_L40_4) or *araC* (in strains S28, IOEB_9805 and IOEB_0501) does not modify the phenotype, suggesting either no link with arabinose metabolism, or the presence of “rescue” genes in the chromosome. In the same way, the absence or mutation of *araT* does not modify the phenotype (strains IOEB_9304, IOEB_C28 and IOEB_C52), which suggests that other arabinose transporters are present. Several candidate genes were identified through sequence analysis: the permeases genes *mfs locus A1*), *mfs1570* (locus C) or *mfs locus D* (Fig. 5)*,* and the distant permease genes *mfs1681 or xylT2* (Fig. 5 and Additional file 1: Table S1). However, genotype to phenotype correlations do not enable determination of which one is really involved in arabinose uptake, probably because several can act as arabinose carriers simultaneously (Fig. 6).

### D-Xylose

**Fig. 7:**
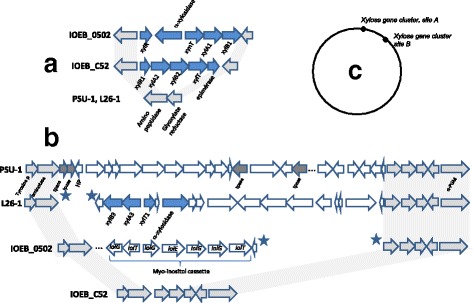
Schematic representation of the 3 xylose gene cassettes of *O. oeni*. **a** Xylose cluster in *O. oeni* IOEB_C52 (GC rate in the cluster 37.2%) and IOEB_0502 (GC: 37.1%) **b** Xylose cluster in *O. oeni* IOEB_L26-1 (GC: 37.9%). **c** Insertion sites of xylose cassettes in *O. oeni* chromosome. *The genes putatively associated with xylose metabolism appear as blue arrows and the dark gray arrows represent genes that may be at the origin of the acquisition of the xylose genes. The stars indicate ends of contigs (draft genomes). XylA: xylulose kinase; XylB xylose isomerase; XylT: permease; XynT xyloside transporteur; XylR: transcription regulator; Tpase: transposase, HP: hypothetical protein, PGM: phosphoglucomutase, aminotase: aminotransferase*

Only three strains (IOEB_0502, IOEB_C52 and IOEB_L26.1) metabolized xylose and they are widely separated on the phylogenomic tree, suggesting no link between phylogeny and phenotype. Three distinct gene cassettes are exclusively found in these 3 strains. Two distinct points of insertion in the chromosome are found (Fig. 7). Despite the absence of GC bias (37.1 to 37.9% GC in the xylose cassettes versus 37.6–38% GC in *O. oeni*), the lack of relationship between the strains suggests a recent acquisition of the cassettes by horizontal gene transfer from distinct donors (probably *Lactobacillus* sp). However, the absence of transposase or phage remnant in the chromosome region surrounding the xylose cassette makes it difficult to identify the mode of acquisition of the genes. The xylose cassettes thus appear not mobile anymore. The 3 cassettes differ by gene content and synteny. The genes *xylA1* and *xylA2* share 97%identity while *xylB1* and *xylB2* were 98% identical. The genes *xylA3* and *xylB3* are more distant (63% identity with *xylA1* and *xylB1* respectively). The two permease genes x*ylT1* and *xylT* share 83% identity and share less than 37% identity with *xynT*. The predicted XylT1 is a highly truncated protein which suggests that other xylose transporters may exist in the chromosome. Indeed, many proteins are annotated as pentose carriers, but correlations do not enable determination of whether one was really involved in xylose uptake. The epimerase gene in the xylose locus of IOEB_C52 is a pseudogene. This and the absence of such a gene in the two other xylose gene clusters suggests that this function is not essential or that a rescue gene exists elsewhere in the chromosome.

### Cellobiose

Cellobiose is β-glucopyranosyl-1,4-D-glucopyranose. The genome survey reveals the presence of several PTS^cel^ permeases and 6-phospho-β-glucosidases genes, grouped into 9 *pts* operons named *celA* to *celI (*Additional file 1: Table S1 and Additional file 5: Figure S5), as well as 3 β-glucosidase genes (*celZ, celZ1* and *celZ2*). Two distinct putative catabolic pathways were thus examined (Additional file 6: Figure S6A). PTS^cel^ activity was measured in *O. oeni* PSU-1, in which *celA* expression was induced by cellobiose [22]. Cellobiose can thus enter the cell and be phosphorylated to cellobiose-6-P before being cut into glucose and glucose-6-P. Meanwhile, all the *celZ* genes encode proteins without a signal peptide. Cellobiose could thus also enter the cell via a permease before being hydrolyzed by CelZ. However, no candidate cellobiose permease could be identified through gene annotation or correlations.

Genotype to phenotype correlations are shown in Additional file 6: Figure S6B. Two strains in branch B display a negative phenotype: S12 and S13. Each one displays a single putatively functional *pts* ^*cel*^ operon. This suggests that *celC* (putatively functional in S13) and *celE* (putatively functional in S12) do not encode cellobiose specific PTS permeases. Moreover, these two negative strains display a potentially functional *celZ* gene. The negative phenotype suggests that in these strains, the cellobiose uptake system (not identified in this study) is not functional. On the other hand, many strains displaying a positive phenotype do not display any entirely functional *pts* ^*cel*^ operon. This suggests that, in these strains, either the route via CelZ and the unidentified permease is functional, or the *pts* ^*cel*^ operons can complement each other.

### Maltose

**Fig. 8:**
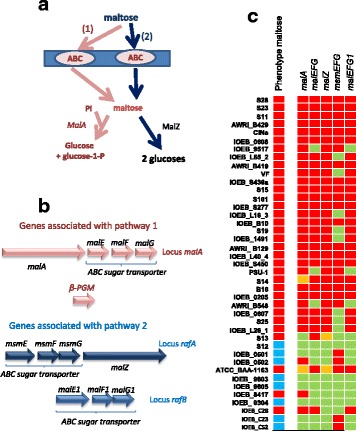
Maltose degradation. **a** Putative maltose specific catabolic pathways. MalA: maltose-phosphorylase, MalZ: alpha-glucosidase, **a**, **b**, **c** sugar permease. Glucose and glucose-1-P then follow the pathways described in Fig. 4. **b** Organization of the genes clusters putatively associated with maltose catabolism. **c** Genotype/phenotype correlations. Strains are listed according to the phylogenomic dendrogram (Additional file 2: Figure S2). In the lane describing the phenotypes, the *blue color* indicates the strains able to grow on maltose as the sole carbon source, and the *red color* indicates the strains unable to grow in such conditions. In the lanes for genotypes, a *red box* indicates that the gene or one of the genes in operons is truncated or appears as a pseudogene. The *green box* indicates no gene truncation but mutations still can lead to inactive proteins. An *orange box* indicates that the corresponding genome sequences displays a specific mutation leading to a singular protein. The β-PGM gene is highly conserved and does not appear in this table

Maltose (α-D-glucopyranosyl-1,4-D-glucopyranose) is metabolized by more than half of the strains in branch B but by none of the strains in branch A (Fig. 1). Two potential maltose catabolic pathways are suggested by gene annotation and analysis (Fig. 8a): a pathway through a maltose-trehalose phosphorylase (locus *malA*), and a pathway involving an intracellular alpha-glucosidase, MalZ (without signal peptide). Three candidates ABC gene clusters are found (*malE t*o *malG*, *msmE* to *msmG* and malE1 to malF1, Fig. 8b). Genotype to phenotype correlations suggest that the cluster *malA* which encodes the maltose-phosphorylase and the first ABC transporter *malEFG* is associated with maltose uptake and degradation (Fig. 8c). However, the strain IOEB_0502 has a positive phenotype despite the truncation of gene *malA*, which suggests that another route exists in this specific strain. The *rafA* and *rafB loci* seem less clearly associated with the metabolism of maltose.

### Melibiose

Melibiose (α-D-galactopyranosyl-1,6-D-glucopyranose) is used as a growth substrate by 78% of the strains studied. All the genome studied display a melibiase gene, *galA*, which appears as a pseudogene in 12 cases. When this gene is not mutated or truncated, a positive phenotype is observed. When it is annotated as pseudogene, the phenotype is negative in 8 cases out of 12. The melibiase sequence displays no signal peptide which suggests that melibiose is first imported into the cell. However, the confrontation of genotypes and phenotype does not enabled identification of the melibiose uptake system. The galactose moiety of melibiose is not used by most of the strains examined and accumulates in the culture medium according to HPLC analysis. The gene encoding the protein allowing the export of galactose was not identified.

### Sucrose

Sucrose (α-D-glucopyranosyl-1,2-β-D-fructofuranose) is used as a growth substrate by 20% of the strains studied, all belonging to branches B and C. Genotype to phenotype correlations indicate that sucrose catabolism is not linked to sucrose phosphorylase (EC.2.4.1.7) Indeed, a gene annotated as encoding a sucrose-phosphorylase is found to belong to the core genome, but it is truncated in 32 of the studied strains, including sucrose positive ones. A non-functional *pts* ^*sucrose*^ operon (with a truncated sucrose-6-P phosphorylase gene, Additional file 1: Table S1) was found in a single strain (ATCC_BAA-1163), but sucrose degradation rather appears to be linked to the presence of a functional levansucrase gene, *levO*. LevO is exocellular and drives the synthesis of levan, a fructan and releases glucose [25]. The growth observed would be due to the uptake and use of the glucose left. However, other pathways may be also active in strains IOEB_0501, IOEB_0502, IOEB_C52 and IOEB_C28 which produce a more intense color change during phenotyping experiments. Nevertheless the correlations did not enable identification of any additional pathway.

### Galactose, raffinose

A galactose cluster with genes whose annotation suggests that it encodes all the functions necessary for uptake, phosphorylation and isomerization of galactose is found in the core genome (Additional file 1: Table S1). However, no correlation clearly associates these genes with galactose phenotype. For raffinose (α-D-galactopyranosyl-1,6-α-D-glucopyranosyl-1,2-β-D-fructofuranose), no link with levansucrase, melibiase or any other glycoside-hydrolase gene was obvious in *O. oeni.*

### Lactose, mannitol, sorbitol, L-rhamnose, and L-sorbose

Lactose, mannitol, sorbitol, L-rhamnose, and L-sorbose were not used as a growth substrate by any the studied strain. For lactose (β-D-galactopyranosyl-1,4-D-glucopyranose), three genes encoding intracellular beta-galactosidase are found and two of them belong to the core genome (*lacZ, lacZ1*, Additional file 1: Table S1), but their role remains unclear. A non-functional *pts* ^*lactose*^ operon is also found in a single strain (IOEB_40.4).

Sorbitol is also not used, despite many genes are annotated as encoding sorbitol dehydrogenase. A non-functional mannitol cassette was found in strain IOEB_C52 (branch C), suggesting that, in *O. oeni* strains others than those studied, mannitol could be transported through a PTS permease and then converted to fructose-6-Phosphate by mannitol-1-P deshydrogenase (Additional file 1: Table S1).

### Additional genes putatively associated with carbohydrate metabolism

Additional genes putatively associated with carbohydrate metabolism were found: (i) a gluconate cluster comprising a permease and a gluconate kinase gene (ii), an ABC transporter putatively associated with 2-deoxy-ribose uptake (iii) a *pts* ^*galactitol*^ operon, *gatA*. According to TCDB database, PTS^galactitol^ is poorly described and its substrate specificity remains unclear. These 3 gene clusters form part of the core genome. Two non-functional myo-inositol cassettes were specifically found in *O. oeni* IOEB_0502 and IOEB_C52 (see Fig. 7 for strain IOEB_C52). Furthermore, genes putatively involved in D-arabinose metabolism (*gutQ*) or L-xylulose (*xylA* and *xylB*) were found (Additional file 1: Table S1). Additionally, a α-mannosidase gene was found in 6 of the studied strains (Additional file 1: Table S1). No amylase or pectinase genes were found in the genome sequences examined.

### Comparison with *O. kitaharae*

According to our results, most of the genes putatively associated with carbohydrate metabolism belong to the core genome. We therefore examined whether these genes or gene clusters are also present in the genome of *O. kitaharae* [27]. A single strain, *O. kitaharae* DSM_17330 was examined (Table 2). The presence of identical gene clusters (regarding gene content and gene synteny) in the same insertion site of the chromosome in both species suggests that these clusters have been inherited from the common ancestor of these two *Oenococcus* species. This is the case of *manB*, *mfs0136*, *nagC*, *nagC1* and *nagC2*, *manI*, *manC*, the fructose cluster, *pgi, pgm, galA* and the gluconate cluster. The positive phenotypes observed in both species when these genes encode putatively active proteins emphasize their putative role in glucose, mannose, fructose/mannitol and melibiose metabolism respectively.

**Table 2:** Comparison with *O. kitaharae* DSM17330

Sugar	*O. oeni* most frequent phenotype^a^	*O. kitaharae* DSM17330 phenotype ^b^	Associated genes identified in *O. oeni* ^c^	Present in *O. kitaharae*?^d^
Glucose	+	+	*manB, manA* *mfs0819, mfs1574*, *mfs0136* *nagC, C1 and C2*	*manB, manA* *mfs136* *nagC, C1 and C2*
Mannose	+	+	*manB, manI*	*manB, manI*
Fructose	+/−	+	*manC (ss), pgi* *ptsfru* (ss), *fruK, fba*, *tpi* Fructose gene cluster	*manC*, *pgi* distinct *ptsfru, fruK, tpi* but *fba* absent Fructose gene cluster
Galactose	−/+	+	Galactose cluster	Galactose cluster with distinct permease gene
Maltose	+/−	+	*malA* cluster	Distinct *malA* cluster
Trehalose	+	+	*treA*	Distinct *treA* cluster
Melibiose	+	+	*galA*	*gala*
Arabinose	+/−	-	Arabinose cluster A	Cluster absent
Ribose	+	-	ribose clusters 1, 2 and 3	cluster ribose1 absent, locus ribose 3 disrupted
Gluconate	ND	weak	gluconate cluster	gluconate cluster
Galactitol	ND	ND	*gatA* cluster	*gatA* absent

^a^this study

^b^according to Endo and Okada [50]

^c^ss: strain specific

^d^ *O. kitaharae* DSM17330 genome sequence [27] was analyzed for the presence of genes putatively associated with the metabolism of a selection of carbohydrate in *O. oeni*. The expression distinct cluster is mentioned if *O. kitaharae* DSM17330 corresponding gene cluster does not display exactly the same gene content (gene identity or synteny) and/or if they are located at distinct insertion sites on the chromosome according to surrounding region

The galactose cluster displays the same insertion site in both species but it is modified in the 5’ end due to the presence of a distinct lactose/galactose permease in *O. kitaharae*. This may at least partly explain the positive galactose phenotype in *O. kitaharae* and the negative one in *O. oeni.* A part of the ribose clusters is absent in *O. kitaharae* and this may explain the ribose negative phenotype.

On the contrary, distinct insertion sites for the same cluster suggest that distinct acquisition events occurred in each species. This is the case for clusters *malA* and *treA*, respectively implicated in maltose and trehalose metabolism but also for the *pts* ^*fru*^ clusters, that may not be active in *O. kitaharae,* due to the absence of *fba*. The arabinose and the *gatA* clusters are specifically present in *O. oeni* and may have been acquired at the species divergence, together with the rescue glucose uptake systems *mfs1574* and *mfs0819*.

### Outcome to correlations

To identify genes and pathways associated with carbohydrate catabolic abilities, two complementary methods were used. Through gene trait matching, we did not get any result that correlated the absence of a gene with a positive trait (growth on a particular sugar). However, this method confirmed genes potentially involved in the metabolism of xylose [24] and identified the ribose permease specifically present in *O. oeni* IOEB_C23. Combining this method with the analysis of mutations inducing singular genes sequence or gene truncations allowed the identification of genes potentially associated with the metabolism of mannose, melibiose, sucrose and maltose. Additional analyzes were required in many other cases, due to probable complementation phenomena (in the case of trehalose, arabinose, ribose) or to multiple metabolic pathways (in the case of glucose, fructose or cellobiose). Siezen et al. [34], Bottaciani et al. [35] and Loux et al. [36] had already raised this problem with other LAB species. Moreover, sugar carriers are very numerous in *O. oeni* [19]. For example, several Na-Melibiose carriers genes are present in *O. oeni* as reported by Unden and Zaunmüller [14], and complementation between them could make the analysis of the correlations difficult. Furthermore, the sequence analysis of protein involved in sugar uptake remains difficult for identifying both the substrate specificity and the mutations making the carrier inactive. Probably for these reasons, we did not identify the metabolic pathways associated with the assimilation of galactose, sucrose and raffinose. Besides, certain phenotypes such as lactose or sucrose were previously described to be unstable [16].

### Importance for *O. oeni* evolution and adaptation

Most of the genes identified belong to the core genome, in agreement with Borneman et al. [20, 24] and they are spread throughout the chromosome with the mosaic distribution described by Klaenhammer et al. [37], and not gathered into a life style cassette as in *Lb. plantarum* [34, 38, 39] or into large genomic islands as capsular exopolysaccharide gene clusters [25]. Moreover, as in other genera [35, 36], the genes are frequently organized in gene clusters encoding two or more functions associated with a specific carbohydrate metabolism, but several distant clusters on the chromosome are often required. This highly conserved scattered chromosomal distribution is generally found in species or subspecies in which all strains share very similar ecological niches and in which no individual evolved to colonize new environments [36, 40, 41]. This may protect the bacteria from losing several carbohydrate degradation abilities through a single recombination event. As proposed for other species [36, 37, 40], the genetic origin of the phenotypes observed in *O. oeni* may include majors events that led to the global evolution of the species (acquisition of the arabinose, trehalose and maltose clusters, and of the rescue glucose uptake systems), but also marginal events, such as acquisition/loss of genes in a limited number of strains (xylose cluster, *pts* ^*fru*^, *manC*, levO) and many punctual mutations that lead to phenotype loss without gene loss. The list of gene clusters conserved or acquired at the emergence of *O. oeni* species suggests that the gradual specialization of *Oenococci* to the wine environment required mainly the maintenance of catabolic activities towards glucose, ribose, trehalose, mannose, cellobiose, melibiose, maltose and arabinose. This is mostly in agreement with the phenotypic results obtained, which also globally match those of literature [2, 5, 13, 15, 16]. The strains in branch A appear more specialized, with reduced abilities compared to strains in branches B and C, which is in agreement with the domestication of strains in Branch A [23, 34]. Branch A gathers several strains that have been selected as malolactic starters but these strains did not display specific catabolic abilities. The metabolism of sucrose and maltose in branch B and C would extend the adaptation of these strains to fruit juices others than grapes.

The ubiquitous glucose consumption can be explained by complementation phenomena between a main pathway and back-up ones, as frequently described in low GC Gram positive bacteria [42]. The main route found in this study is via the permease ManB, generally described as associated with catabolic repression [43, 44]. Glucose would thus be the favorite substrate *O. oeni* in grapes musts, where it displays very high concentrations (>150 g/l). Catabolic repression in wine may be less strong thanks to the low glucose concentrations often observed (<2 g/l, 9).

The high proportion of strains growing on ribose, trehalose or mannose as the sole substrate reflects the adaptation to development in wine at the end of alcoholic fermentation (liberation of trehalose and mannoproteins from yeast lysates, 9).

Besides, the ability to use substrates coming from grapes, especially melibiose and arabinose, is quite common in *O. oeni*. Cellobiose is also very often consumed and this phenotype may reflect the ability to use the beta-glucosides found in grapes. This ability is ubiquitous in branch A while the degradation of melibiose and arabinose are more common in branch B. Arabinose degradation was proposed as a classification base for wine lactic acid bacteria [13]. We show that this phenotype is partially related to the phylogeny. For xylose, several gene cassettes acquired by HGT were highlighted in a low number of strains [24] and we obtained good correlations with phenotypes. This shows that the proposition by Peynaud and Domercq [13] to use xylose metabolism as an additional strain classification element has no genetic basis, although it remains interesting from a physiological and technological point of view (pentose metabolism leads to the formation of increased amounts of acetic acid in wines).

The main disagreement between our phenotype results and previous reports lies in the metabolism of fructose. In our study, less than 50% of the strains effectively metabolized fructose, while it is often described as ubiquitous [5, 14]. Several studies described metabolite (lactate and acetate) production yields compatible with the route (i) through ManC or (ii) through permease + NagC [6, 8, 45]. Genotype to phenotype correlation did not enable a conclusion on the role of this second pathway. Nevertheless, it was the pathway proposed by Salou et al. [6] and it would be the only compatible with the growth of 4 of the studied strains. However, there is no element to explain why this pathway would be inactive in most strains displaying a fructose negative phenotype. This pathway could be active but very slow in the negative strains. This would be consistent with the fact that Salou et al. [6] indicate a loss of energy during growth on fructose much higher than on glucose. This energy loss was also observed with *Leuconostoc mesenteroides* [46]. On the other hand, the route through PTS^fru^ and homolactic fermentation is completely new in *O. oeni* and, in strains that display it, fructose may be preferred to glucose in winemaking situations, as homolactic fermentation is much more profitable from the energy point of view. This will be compatible with the preference for fructose described by Silvano et al. [47] but not obtained by others [6, 8]. Such odd metabolic pathways are described for fructose uptake and degradation in specific strains in other species [48, 49].

## Conclusions

Through our study, we were able to list the main sugars used as growth substrates by *O. oeni* and to propose or confirm putatively associated metabolic pathways. *O. oeni* appears as a highly specialized species, ideally suited to fermented fruit juice for strains in branches B and C, and more specialized to wine for strains in branch A. The analysis of genes and associated metabolic pathways allowed speculation on the age of the metabolic pathways in the *Oenococcus* genus and in the species *O. oeni*, but also on the substrate preferences that could be observed in the winemaking situation. Analyses of carbohydrates concentrations in wines throughout the winemaking process will be interesting to verify these hypotheses in the future.

## Additional files

Additional file 1: Table S1. Protein sequences database. (XLSX 92 kb)
Additional file 2: Figure S2. Phylogenomic relationship between the strains studied according to dendrogram reconstruction by ANIm. The major genetic groups are indicated (Branch A, B or C). Strains coming from the same type of wine (Champagne, cider) are indicated when they form a single cluster. *Adapted from* [23]. (PPTX 66 kb)
Additional file 3: Figure S3. Conservation of the fructose cluster in *Leuconostoc mesenteroides, Lactobacillus brevis* and *Oenococci.* Mtd in *O. oeni* PSU-1 displays 91% identity with that of *O. kitaharae* DSM 17330, 87% identity with that of *O. alcoholitolerans* CBAS474*,* 79% identity with that of *Lb. brevis* ATCC367 and 73% identity with that of *L. mesenteroides* ATCC 8293*.* NagC1: hexokinase; Mtd: mannitol deshydrogenase, Ycze: MFS permease. (PPTX 63 kb)
Additional file 4: Figure S4. Ribose degradation. A. Putative pathway for ribose transport, phosphorylation and hydrolysis. The co substrates such as ATP are not indicated. B. Organization of the different genes clusters putatively associated with ribose catabolism. C. Genotype/phenotype correlations. Strains appear in the same order as on the phylogenomic dendrogram (Figure S1). In the lane describing the phenotypes, the blue color indicates the strains able to grow on ribose as the sole carbon source, and the red color indicates the strains unable to grow in such conditions. In the lanes for genotypes, a beige box indicates that the gene or the operon is absent. A red box indicates that the gene or one of the genes in operons is truncated or appears as a pseudogene. The green color indicates no gene truncation but mutations still can lead to inactive proteins. An orange box indicates that the corresponding genome sequence displays a specific mutation leading to a singular protein. (PPTX 88 kb)
Additional file 5: Figure S5. The distinct PTS cellobiose operons and their insertion sites on *O. oeni* chromosome. The blue arrows represent the genes in the *pts* ^*cel*^ operons, and the gray arrows represent adjacent genes: HP hypothetical protein, 6-P-β-gluc: 6-Phospho-beta-glucosidase; IIA IB, IIC or IIABC: elements of the PTS permease; regul: transcription regulator. *O. oeni* PSU-1 is represented with its own *pts* ^*cel*^ clusters (A B, C, D …), and the adjacent regions of others *pts* ^*cel*^ clusters enabled to localize them. (PPTX 71 kb)
Additional file 6: Figure S6. Cellobiose degradation. A. Putative cellobiose catabolic pathways. A and IIBC: components of the cellobiose PTS permease, BglB: 6-phospho-beta-glucosidase, CelZ: beta glucosidase. B. Genotype/phenotype correlations. Strains are listed according to the phylogenomic dendrogram (Additionnal file 2: Figure S2). In the lane for phenotypes, the blue boxes indicate the strains able to grow on cellobiose as the sole carbon source, and the red ones indicate the strains unable to grow in such conditions. In the lanes for genotypes, a beige box indicates that the gene or the gene cluster is absent. A red box indicates that the gene (or one of the genes in the *pts* operon) is truncated or appears as a pseudogene. The green color indicates no gene truncation but mutations still can lead to inactive proteins. (PPTX 96 kb)

## Abbreviations

AraA: L-arabinose isomerase
AraB: L-ribulokinase
AraC: Transcription regulator
AraD: L-ribulose-5-P-isomerase
AraT: Arabinose permease
CelA, CelB, CelC, CelD, CelE, CelF, CelG, CelH, CelI: Cellobiose specific PTS permease
CelZ: Beta-glucosidase
Cfu: Colony forming unit
Fba: Fructose-biphosphate alsolase
GatA: Melibiase
HPLC: High performance liquid chromatography
LacZ: Beta-galactosidase
LevO: Levansucrase
MalA: Maltose/trehalose phosphorylase
MalEFG: ABC transporter
ManA, ManB, ManC: Mannose specific PTS permease
ManI: Phosphomannose isomerase
MFS: Mfs, major facilitator system (transport proteins)
MsmEFG: ABC transporter
Mtd: Mannitol deshydrogenase
NagC: Hexokinase
OD: Optical density
Pgi: Phospho-glucose isomerase
Pgm: Phosphoglucomutase
PTS: Pts, phosphotransferase system (sugar specific uptake system)
RibA: Ribose-5-P-isomerase
RibK: Ribokinase
RibT: Ribose permease
SMD: Semi defined medium
TreA: Trehalose specific PTS permease
TreC: Trehalose -6-P hydrolase
Ycze: MFS permease
