# Supplementary material for: Carbohydrate metabolism in Oenococcus oeni: a genomic insight
Source: BMC Genomics. 2016 Dec 1;17:984. doi: 10.1186/s12864-016-3338-2 (PMC5131533; doi:10.1186/s12864-016-3338-2)
Supplement: Additional file 2: Figure S2. — Phylogenomic relationship between the strains studied according to dendrogram reconstruction by ANIm. The major genetic groups are indicated (Branch A, B or C). Strains coming from the same type of wine (Champagne, cider) are indicated when they form a single cluster. Adapted from [23]. (PPTX 66 kb) [file 12864_2016_3338_MOESM2_ESM.pptx]

## Slide 1
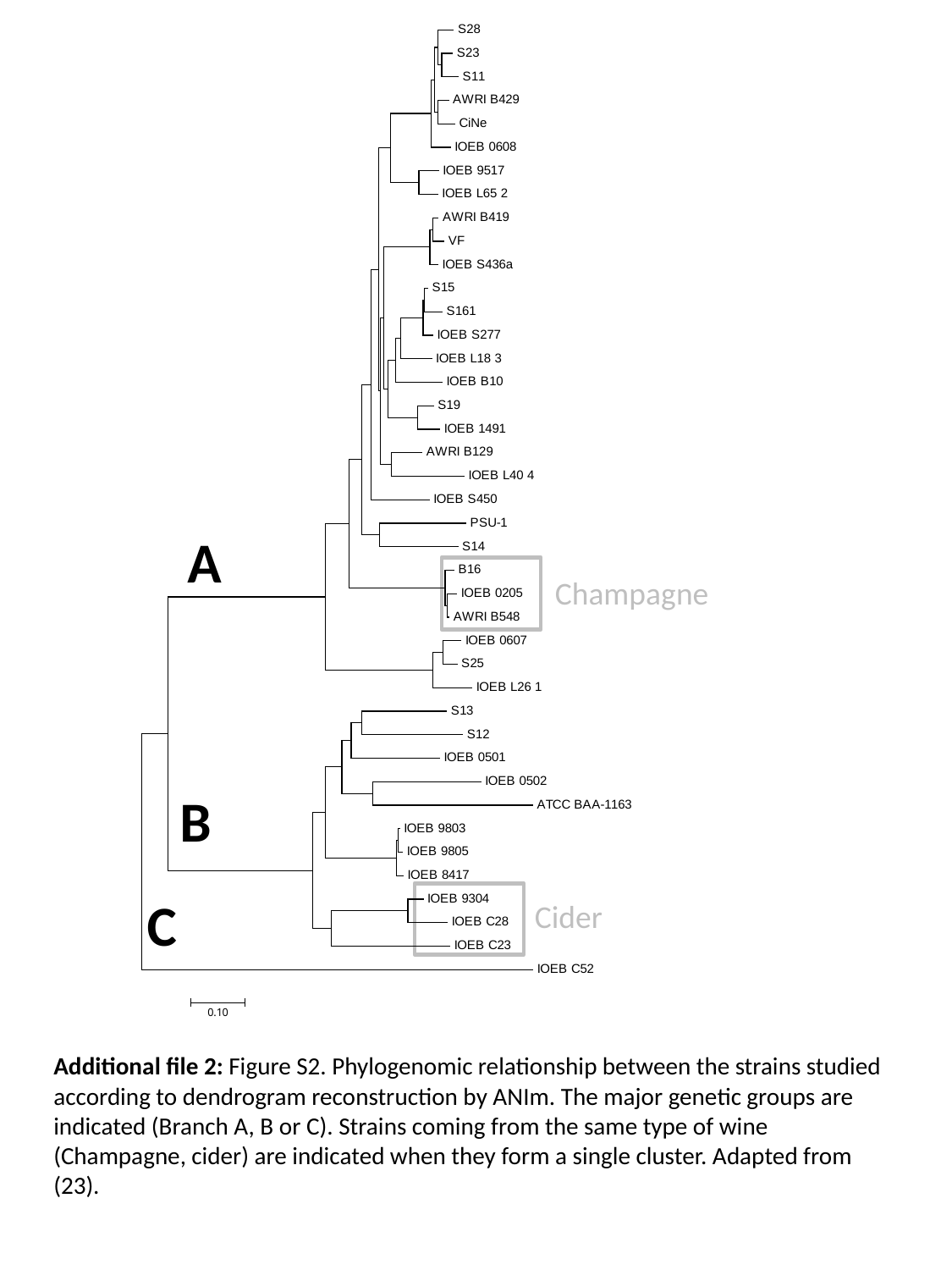

A
Champagne
B
C
Cider
Additional file 2: Figure S2. Phylogenomic relationship between the strains studied according to dendrogram reconstruction by ANIm. The major genetic groups are indicated (Branch A, B or C). Strains coming from the same type of wine (Champagne, cider) are indicated when they form a single cluster. Adapted from (23).
