# Supplementary material for: Carbohydrate metabolism in Oenococcus oeni: a genomic insight
Source: BMC Genomics. 2016 Dec 1;17:984. doi: 10.1186/s12864-016-3338-2 (PMC5131533; doi:10.1186/s12864-016-3338-2)
Supplement: Additional file 3: Figure S3. — Conservation of the fructose cluster in Leuconostoc mesenteroides, Lactobacillus brevis and Oenococci. Mtd in O. oeni PSU-1 displays 91% identity with that of O. kitaharae DSM 17330, 87% identity with that of O. alcoholitolerans CBAS474, 79% identity with that of Lb. brevis ATCC367 and 73% identity with that of L. mesenteroides ATCC 8293. NagC1: hexokinase; Mtd: mannitol deshydrogenase, Ycze: MFS permease. (PPTX 63 kb) [file 12864_2016_3338_MOESM3_ESM.pptx]

## Slide 1
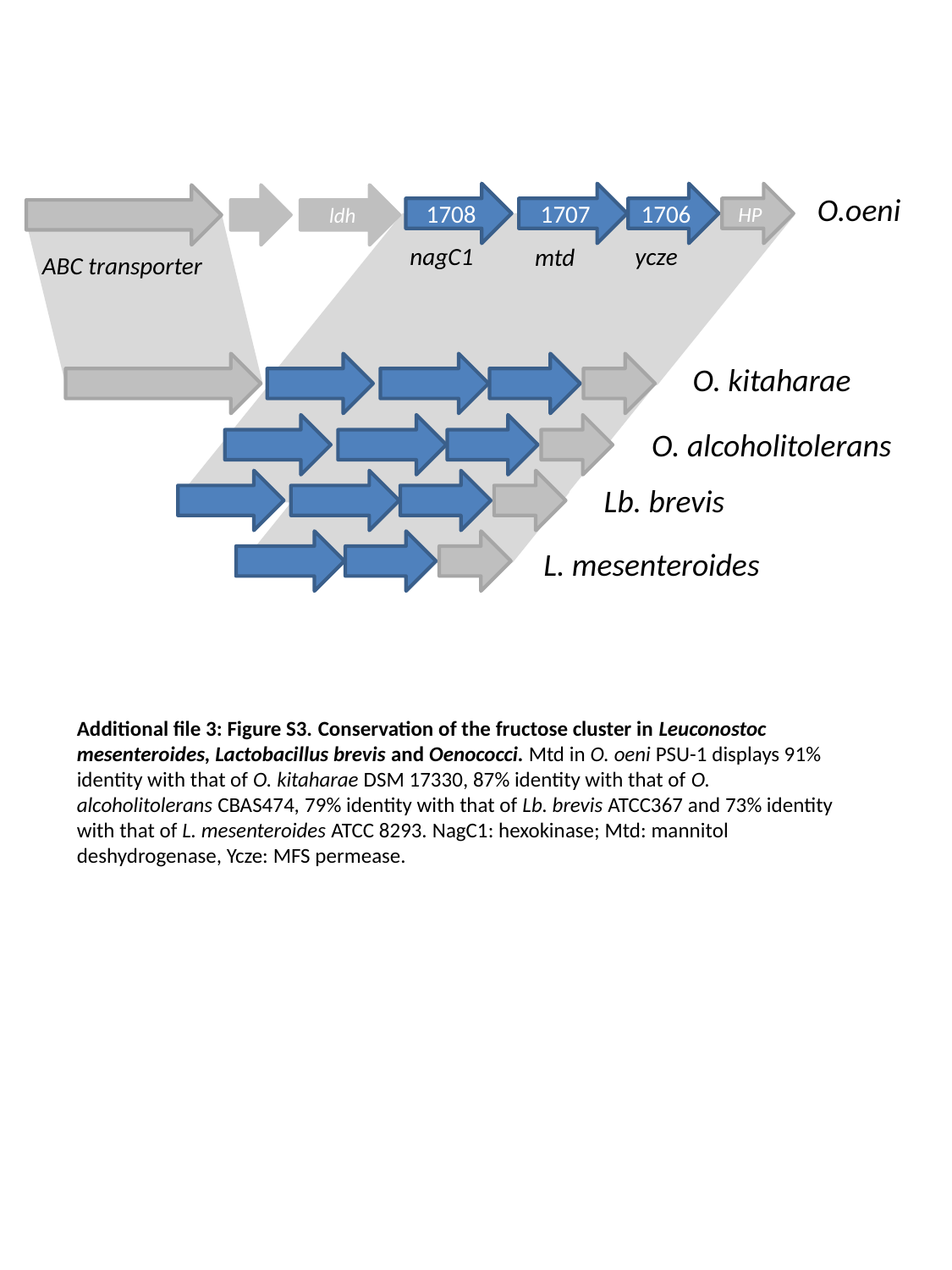

O.oeni
1708
1707
1706
HP
ldh
nagC1
ycze
mtd
ABC transporter
O. kitaharae
O. alcoholitolerans
Lb. brevis
L. mesenteroides
Additional file 3: Figure S3. Conservation of the fructose cluster in Leuconostoc mesenteroides, Lactobacillus brevis and Oenococci. Mtd in O. oeni PSU-1 displays 91% identity with that of O. kitaharae DSM 17330, 87% identity with that of O. alcoholitolerans CBAS474, 79% identity with that of Lb. brevis ATCC367 and 73% identity with that of L. mesenteroides ATCC 8293. NagC1: hexokinase; Mtd: mannitol deshydrogenase, Ycze: MFS permease.
