# Supplementary material for: Carbohydrate metabolism in Oenococcus oeni: a genomic insight
Source: BMC Genomics. 2016 Dec 1;17:984. doi: 10.1186/s12864-016-3338-2 (PMC5131533; doi:10.1186/s12864-016-3338-2)
Supplement: Additional file 4: Figure S4. — Ribose degradation. A. Putative pathway for ribose transport, phosphorylation and hydrolysis. The co substrates such as ATP are not indicated. B. Organization of the different genes clusters putatively associated with ribose catabolism. C. Genotype/phenotype correlations. Strains appear in the same order as on the phylogenomic dendrogram (Figure S1). In the lane describing the phenotypes, the blue color indicates the strains able to grow on ribose as the sole carbon source, and the red color indicates the strains unable to grow in such conditions. In the lanes for genotypes, a beige box indicates that the gene or the operon is absent. A red box indicates that the gene or one of the genes in operons is truncated or appears as a pseudogene. The green color indicates no gene truncation but mutations still can lead to inactive proteins. An orange box indicates that the corresponding genome sequence displays a specific mutation leading to a singular protein. (PPTX 88 kb) [file 12864_2016_3338_MOESM4_ESM.pptx]

## Slide 1
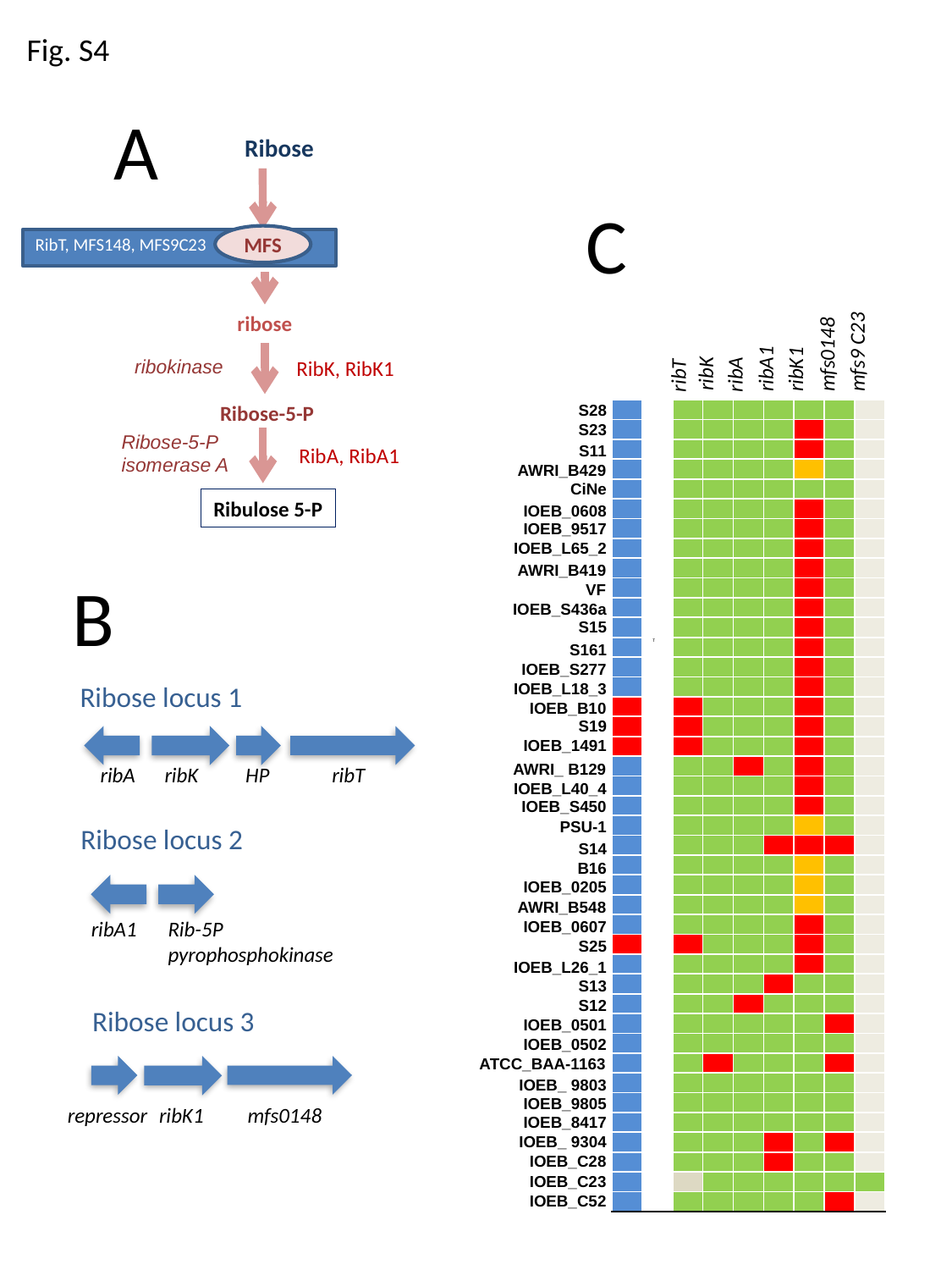

Fig. S4
A
Ribose
C
MFS
RibT, MFS148, MFS9C23
ribK
ribose
mfs9 C23
mfs0148
ribokinase
RibK, RibK1
ribA1
ribK1
ribA
ribT
Ribose-5-P
| | | | | | | | | |
| --- | --- | --- | --- | --- | --- | --- | --- | --- |
| | | | | | | | | |
| | | | | | | | | |
| | | | | | | | | |
| | | | | | | | | |
| | | | | | | | | |
| | | | | | | | | |
| | | | | | | | | |
| | | | | | | | | |
| | | | | | | | | |
| | | | | | | | | |
| | T | | | | | | | |
| T | T | | | | | | | |
| | T | | | | | | | |
| | T | | | | | | | |
| | | | | | | | | |
| | - | | | | | | | |
| | - | | | | | | | |
| | - | | | | | | | |
| | - | | | | | | | |
| | | | | | | | | |
| | | | | | | | | |
| | - | | | | | | | |
| | | | | | | | | |
| | - | | | | | | | |
| | | | | | | | | |
| | | | | | | | | |
| | | | | | | | | |
| | | | | | | | | |
| | | | | | | | | |
| | | | | | | | | |
| | | | | | | | | |
| | | | | | | | | |
| | | | | | | | | |
| | | | | | | | | |
| | | | | | | | | |
| | | | | | | | | |
| | | | | | | | | |
| | | | | | | | | |
| | | | | | | | | |
| | | | | | | | | |
 S28
S23
 S11
 AWRI_B429
 CiNe
 IOEB_0608
 IOEB_9517
 IOEB_L65_2
 AWRI_B419
 VF
 IOEB_S436a
 S15
 S161
 IOEB_S277
 IOEB_L18_3
 IOEB_B10
 S19
 IOEB_1491
AWRI_ B129
 IOEB_L40_4
 IOEB_S450
 PSU-1
 S14
 B16
 IOEB_0205
 AWRI_B548
 IOEB_0607
 S25
 IOEB_L26_1
 S13
 S12
 IOEB_0501
 IOEB_0502
ATCC_BAA-1163
IOEB_ 9803
 IOEB_9805
 IOEB_8417
IOEB_ 9304
 IOEB_C28
 IOEB_C23
 IOEB_C52
Ribose-5-P
isomerase A
RibA, RibA1
Ribulose 5-P
B
Ribose locus 1
ribA
ribK
HP
ribT
Ribose locus 2
ribA1
Rib-5P pyrophosphokinase
Ribose locus 3
repressor
ribK1
mfs0148

## Slide 2
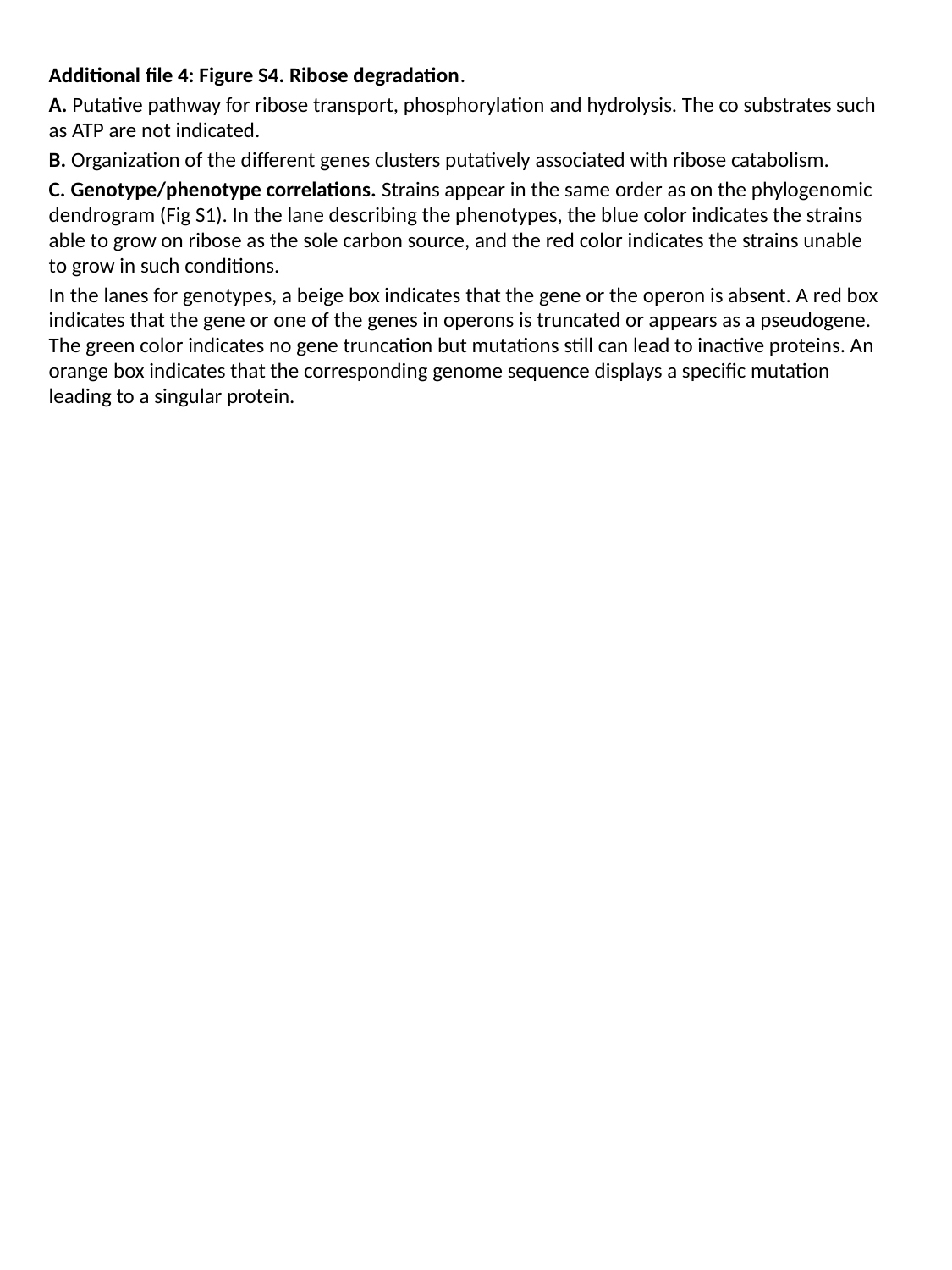

Additional file 4: Figure S4. Ribose degradation.
A. Putative pathway for ribose transport, phosphorylation and hydrolysis. The co substrates such as ATP are not indicated.
B. Organization of the different genes clusters putatively associated with ribose catabolism.
C. Genotype/phenotype correlations. Strains appear in the same order as on the phylogenomic dendrogram (Fig S1). In the lane describing the phenotypes, the blue color indicates the strains able to grow on ribose as the sole carbon source, and the red color indicates the strains unable to grow in such conditions.
In the lanes for genotypes, a beige box indicates that the gene or the operon is absent. A red box indicates that the gene or one of the genes in operons is truncated or appears as a pseudogene. The green color indicates no gene truncation but mutations still can lead to inactive proteins. An orange box indicates that the corresponding genome sequence displays a specific mutation leading to a singular protein.
