# Supplementary material for: Carbohydrate metabolism in Oenococcus oeni: a genomic insight
Source: BMC Genomics. 2016 Dec 1;17:984. doi: 10.1186/s12864-016-3338-2 (PMC5131533; doi:10.1186/s12864-016-3338-2)
Supplement: Additional file 5: Figure S5. — The distinct PTS cellobiose operons and their insertion sites on O. oeni chromosome. The blue arrows represent the genes in the pts cel operons, and the gray arrows represent adjacent genes: HP hypothetical protein, 6-P-β-gluc: 6-Phospho-beta-glucosidase; IIA IB, IIC or IIABC: elements of the PTS permease; regul: transcription regulator. O. oeni PSU-1 is represented with its own pts cel clusters (A B, C, D …), and the adjacent regions of others pts cel clusters enabled to localize them. (PPTX 71 kb) [file 12864_2016_3338_MOESM5_ESM.pptx]

## Slide 1
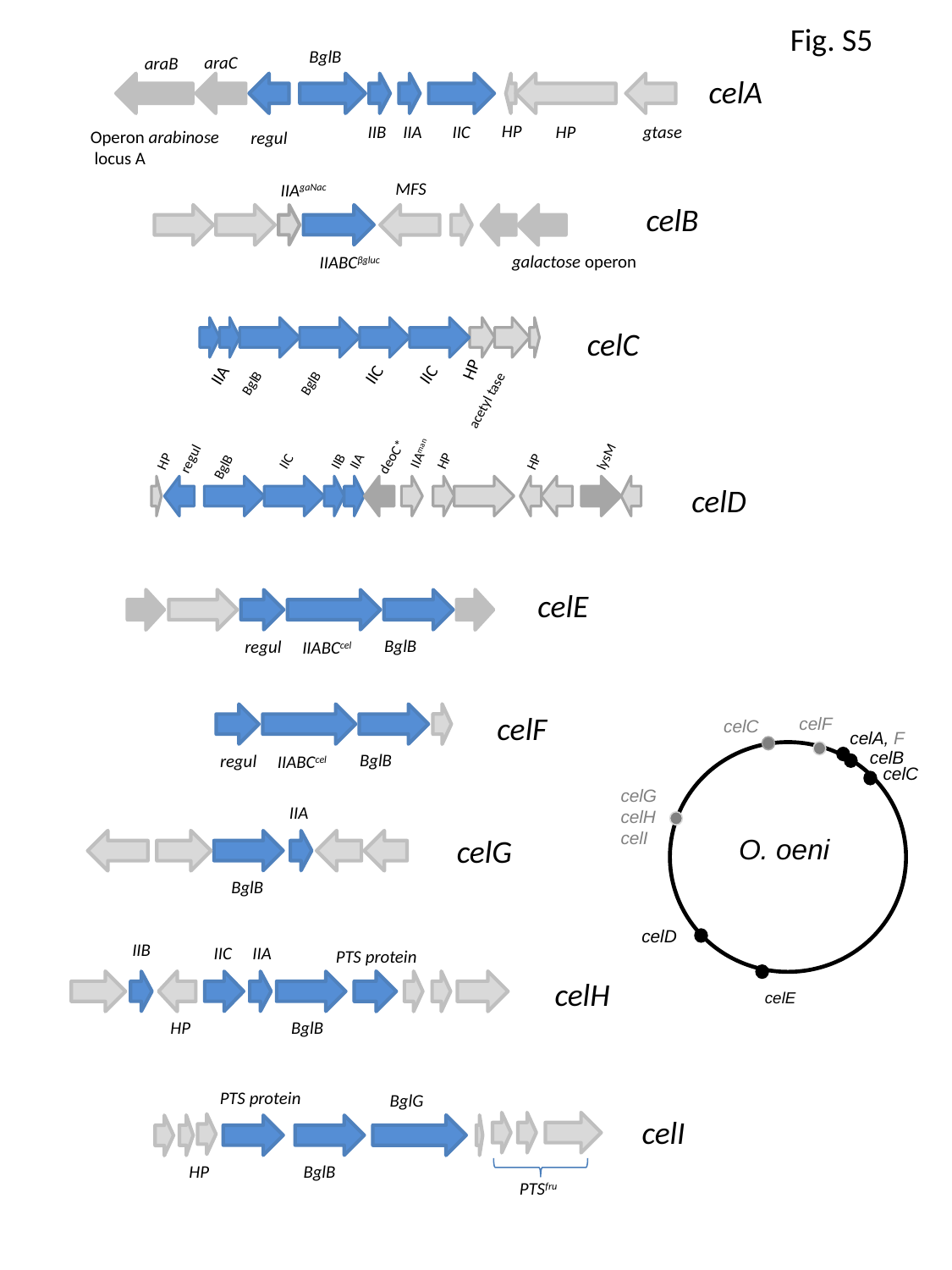

Fig. S5
BglB
araC
araB
celA
HP
IIB
IIA
IIC
HP
gtase
Operon arabinose
 locus A
regul
MFS
IIAgaNac
celB
galactose operon
IIABCβgluc
celC
HP
IIC
IIC
IIA
BglB
BglB
acetyl tase
BglB
IIC
lysM
IIAman
deoC*
regul
HP
HP
IIB
IIA
HP
celD
celE
BglB
regul
IIABCcel
celF
celF
celC
celA, F
celB
BglB
regul
IIABCcel
celC
celG
celH
celI
IIA
celG
O. oeni
BglB
celD
IIB
IIA
IIC
PTS protein
celH
celE
BglB
HP
PTS protein
BglG
celI
BglB
HP
PTSfru

## Slide 2
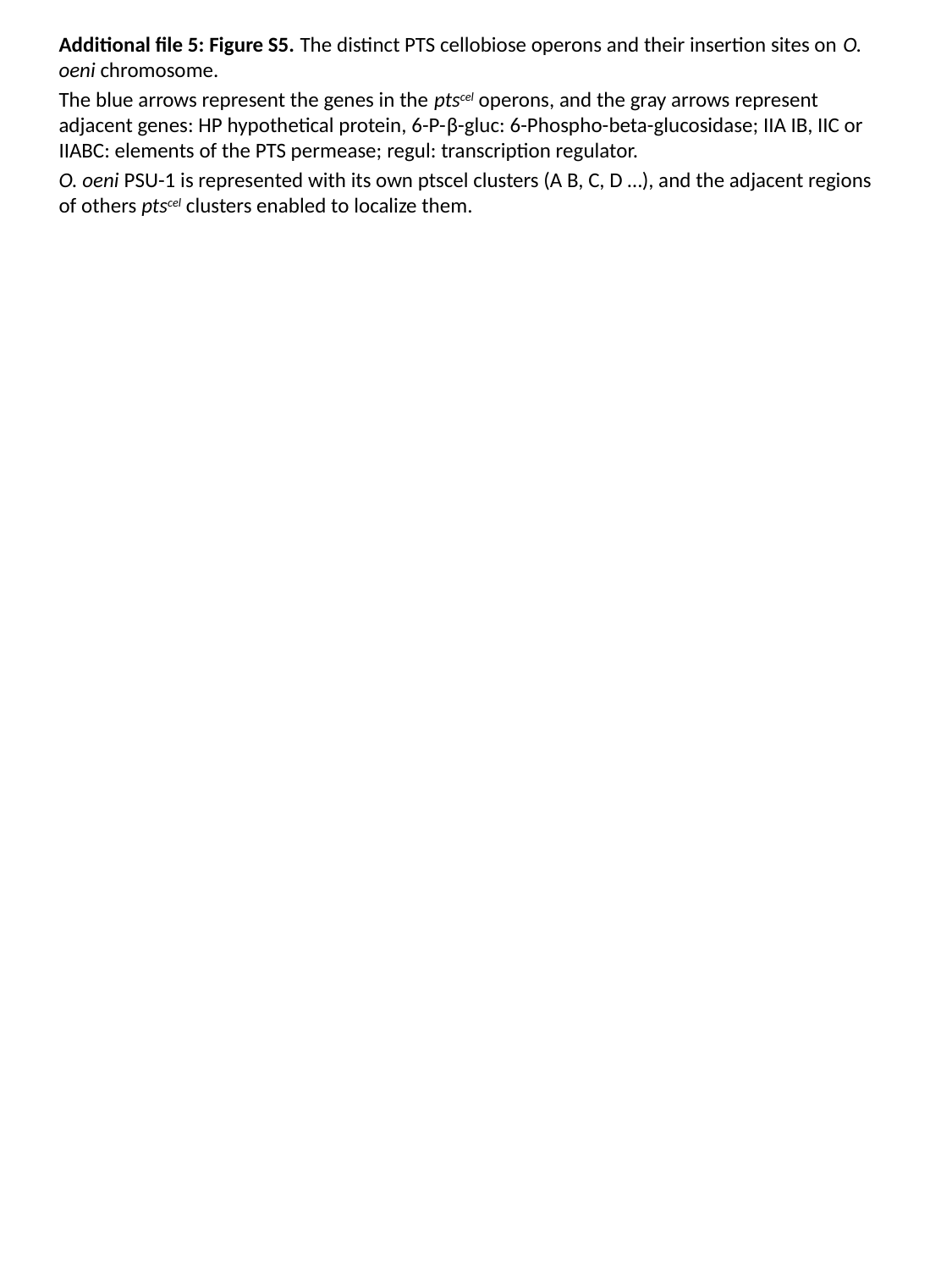

Additional file 5: Figure S5. The distinct PTS cellobiose operons and their insertion sites on O. oeni chromosome.
The blue arrows represent the genes in the ptscel operons, and the gray arrows represent adjacent genes: HP hypothetical protein, 6-P-β-gluc: 6-Phospho-beta-glucosidase; IIA IB, IIC or IIABC: elements of the PTS permease; regul: transcription regulator.
O. oeni PSU-1 is represented with its own ptscel clusters (A B, C, D …), and the adjacent regions of others ptscel clusters enabled to localize them.
