# Supplementary material for: Carbohydrate metabolism in Oenococcus oeni: a genomic insight
Source: BMC Genomics. 2016 Dec 1;17:984. doi: 10.1186/s12864-016-3338-2 (PMC5131533; doi:10.1186/s12864-016-3338-2)
Supplement: Additional file 6: Figure S6. — Cellobiose degradation. A. Putative cellobiose catabolic pathways. A and IIBC: components of the cellobiose PTS permease, BglB: 6-phospho-beta-glucosidase, CelZ: beta glucosidase. B. Genotype/phenotype correlations. Strains are listed according to the phylogenomic dendrogram (Additionnal file 2: Figure S2). In the lane for phenotypes, the blue boxes indicate the strains able to grow on cellobiose as the sole carbon source, and the red ones indicate the strains unable to grow in such conditions. In the lanes for genotypes, a beige box indicates that the gene or the gene cluster is absent. A red box indicates that the gene (or one of the genes in the pts operon) is truncated or appears as a pseudogene. The green color indicates no gene truncation but mutations still can lead to inactive proteins. (PPTX 96 kb) [file 12864_2016_3338_MOESM6_ESM.pptx]

## Slide 1
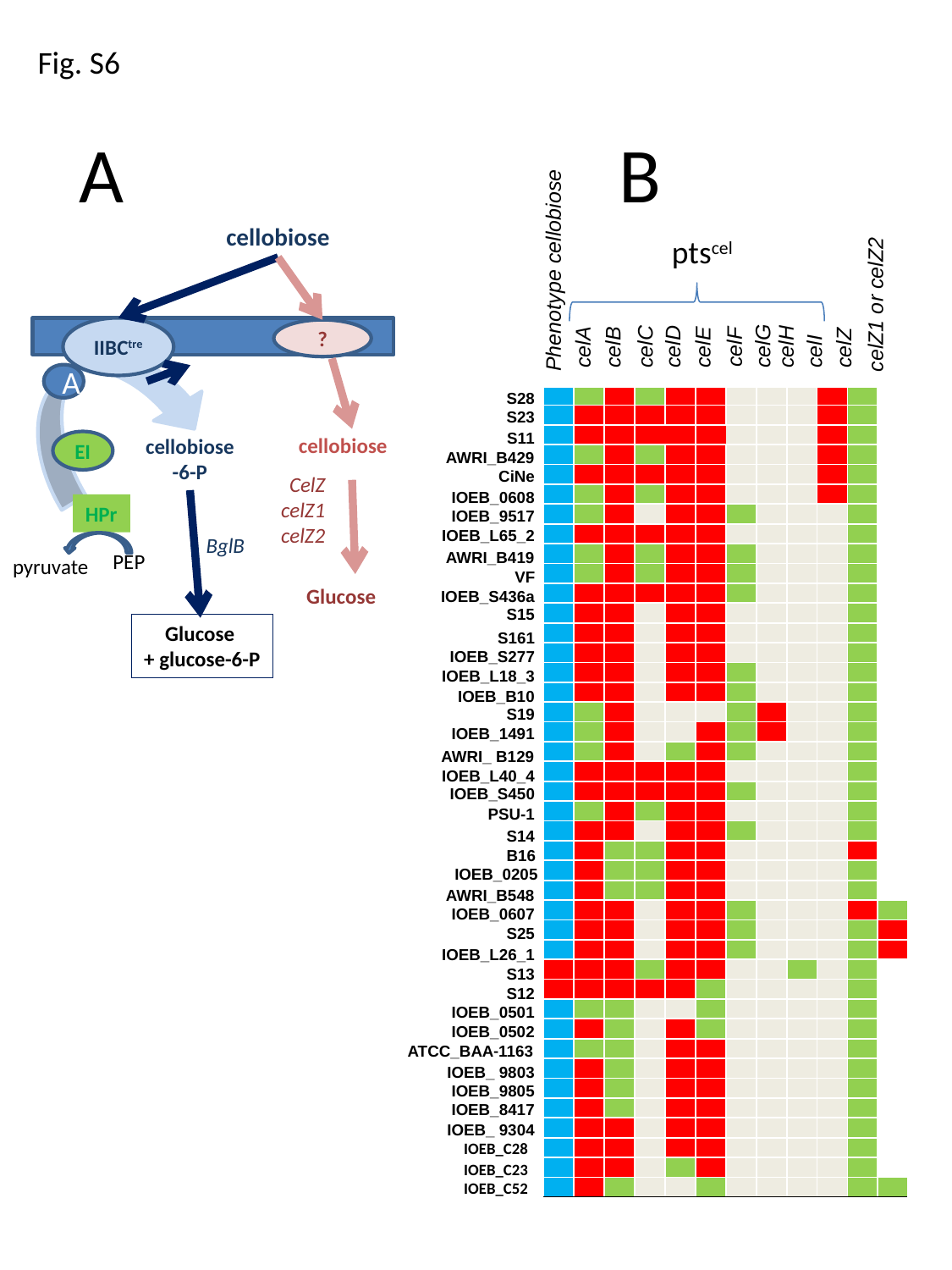

Fig. S6
A
B
cellobiose
IIBCtre
?
A
cellobiose
cellobiose
-6-P
EI
CelZ
celZ1
celZ2
HPr
BglB
PEP
pyruvate
Glucose
Glucose
+ glucose-6-P
ptscel
Phenotype cellobiose
celZ1 or celZ2
celG
celC
celD
celH
celA
celB
celE
celF
celZ
celI
| | | | | | | | | | | | | |
| --- | --- | --- | --- | --- | --- | --- | --- | --- | --- | --- | --- | --- |
| | | | | | | | | | | | | |
| | | | | | | | | | | | | |
| | | | | | | | | | | | | |
| | | | | | | | | | | | | |
| | | | | | | | | | | | | |
| | | | | | | | | | | | | |
| | | | | | | | | | | | | |
| | | | | | | | | | | | | |
| | | | | | | | | | | | | |
| | | | | | | | | | | | | |
| | | | | | | | | | | | | |
| T | | | | | | | | | | | | |
| | | | | | | | | | | | | |
| | | | | | | | | | | | | |
| | | | | | | | | | | | | |
| | | | | | | | | | | | | |
| | | | | | | | | | | | | |
| | | | | | | | | | | | | |
| | | | | | | | | | | | | |
| | | | | | | | | | | | | |
| | | | | | | | | | | | | |
| | | | | | | | | | | | | |
| | | | | | | | | | | | | |
| | | | | | | | | | | | | |
| | | | | | | | | | | | | |
| | | | | | | | | | | | | |
| | | T | | | | | | | | | | |
| | | T | | | | | | | | | | |
| | | T | | | | | | | | | | |
| | | - | | | | | | | | | | |
| | | | | | | | | | | | | |
| | | | T | | | | | | | | | |
| | | | | | | | | | | | | |
| | | | | | | | | | | | | |
| | | | | | | | | | | | | |
| | | | | | | | | | | | | |
| | | | T | | | | | | | | | |
| | | | T | | | | | | | | | |
| | | | | | | | | | | | | |
| | | | | | | | | | | | | |
 S28
 S23
 S11
 AWRI_B429
 CiNe
 IOEB_0608
 IOEB_9517
 IOEB_L65_2
 AWRI_B419
 VF
 IOEB_S436a
 S15
 S161
 IOEB_S277
 IOEB_L18_3
 IOEB_B10
 S19
 IOEB_1491
AWRI_ B129
 IOEB_L40_4
 IOEB_S450
 PSU-1
 S14
 B16
 IOEB_0205
 AWRI_B548
 IOEB_0607
 S25
 IOEB_L26_1
 S13
 S12
 IOEB_0501
 IOEB_0502
ATCC_BAA-1163
IOEB_ 9803
 IOEB_9805
 IOEB_8417
IOEB_ 9304
 IOEB_C28
 IOEB_C23
 IOEB_C52

## Slide 2
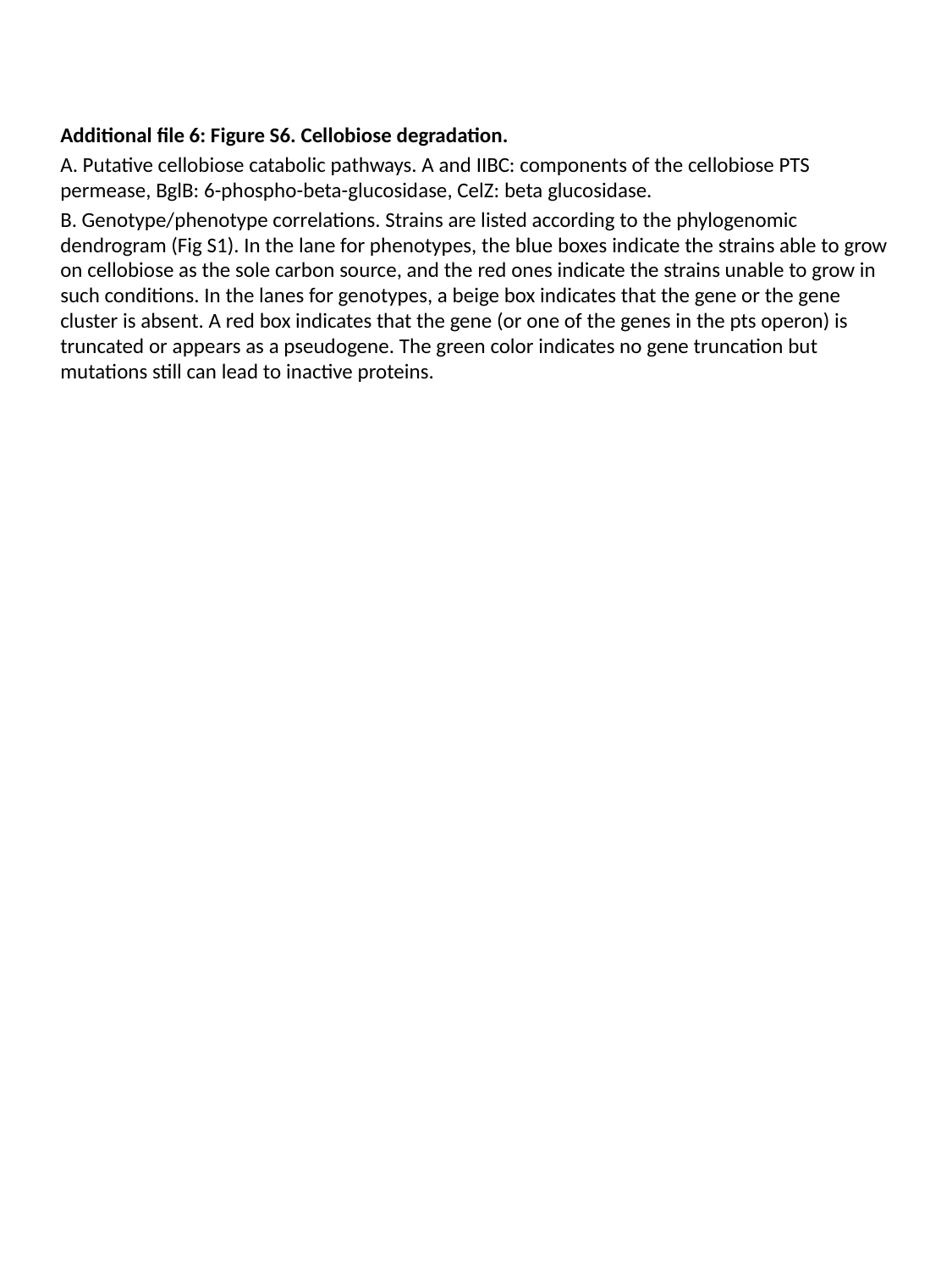

Additional file 6: Figure S6. Cellobiose degradation.
A. Putative cellobiose catabolic pathways. A and IIBC: components of the cellobiose PTS permease, BglB: 6-phospho-beta-glucosidase, CelZ: beta glucosidase.
B. Genotype/phenotype correlations. Strains are listed according to the phylogenomic dendrogram (Fig S1). In the lane for phenotypes, the blue boxes indicate the strains able to grow on cellobiose as the sole carbon source, and the red ones indicate the strains unable to grow in such conditions. In the lanes for genotypes, a beige box indicates that the gene or the gene cluster is absent. A red box indicates that the gene (or one of the genes in the pts operon) is truncated or appears as a pseudogene. The green color indicates no gene truncation but mutations still can lead to inactive proteins.
